# The emergence of genetic variants linked to brain and cognitive traits in human evolution

**DOI:** 10.1093/cercor/bhaf127

**Published:** 2025-08-13

**Authors:** Ilan Libedinsky, Yongbin Wei, Christiaan de Leeuw, James K Rilling, Danielle Posthuma, Martijn P van den Heuvel

**Affiliations:** Department of Complex Trait Genetics, Center for Neurogenomics and Cognitive Research, Amsterdam Neuroscience, Vrije Universiteit Amsterdam, De Boelelaan 1105, Amsterdam 1081HV, the Netherlands; Department of Complex Trait Genetics, Center for Neurogenomics and Cognitive Research, Amsterdam Neuroscience, Vrije Universiteit Amsterdam, De Boelelaan 1105, Amsterdam 1081HV, the Netherlands; School of Artificial Intelligence, Beijing University of Posts and Telecommunications, 10 Xitucheng Road, Beijing 100876, China; Department of Complex Trait Genetics, Center for Neurogenomics and Cognitive Research, Amsterdam Neuroscience, Vrije Universiteit Amsterdam, De Boelelaan 1105, Amsterdam 1081HV, the Netherlands; Emory National Primate Research Center, Emory University, 954 Gatewood Rd NE, Atlanta, GA 30329, United States; Center for Translational Social Neuroscience, Emory University, 55 Park Pl NE, Atlanta, GA 30322, United States; Department of Psychology, Emory University, 36 Eagle Row, Atlanta, GA 30322, United States; Department of Psychiatry and Behavioral Sciences, Emory University, 12 Executive Park Dr NE #200, Atlanta, GA 30322, United States; Department of Complex Trait Genetics, Center for Neurogenomics and Cognitive Research, Amsterdam Neuroscience, Vrije Universiteit Amsterdam, De Boelelaan 1105, Amsterdam 1081HV, the Netherlands; Department of Child and Adolescent Psychiatry and Psychology, Section Complex Trait Genetics, Amsterdam Neuroscience, Vrije Universiteit Medical Center, Amsterdam UMC, De Boelelaan 1105, Amsterdam 1081HV, the Netherlands; Department of Complex Trait Genetics, Center for Neurogenomics and Cognitive Research, Amsterdam Neuroscience, Vrije Universiteit Amsterdam, De Boelelaan 1105, Amsterdam 1081HV, the Netherlands; Department of Child and Adolescent Psychiatry and Psychology, Section Complex Trait Genetics, Amsterdam Neuroscience, Vrije Universiteit Medical Center, Amsterdam UMC, De Boelelaan 1105, Amsterdam 1081HV, the Netherlands

**Keywords:** brain, evolution, genetics, neuropsychiatry, Paleogenomics

## Abstract

Human evolution involved major anatomical transformations, including a rapid increase in brain volume over the last 2 million years. Examination of fossil records provides insight into these physical changes but offers limited information on the evolution of brain function and cognition. A complementary approach integrates genome dating from the Human Genome Dating Project with genome-wide association studies to trace the emergence of genetic variants linked to human traits over 5 million years. We find that genetic variants underlying cortical morphology (~300,000 years, *P* = 4 × 10^−28^), fluid intelligence (~500,000 years, *P* = 1.4 × 10^−4^), and psychiatric disorders (~475,000 years, *P* = 5.9 × 10^−33^) emerged relatively recently in hominin evolution. Among psychiatric phenotypes, variants associated with depression (~24,000 years, *P* = 1.6 × 10^−4^) and alcoholism-related traits (~40,000 years, *P* = 5.2 × 10^−12^) are the youngest. Genes with recent evolutionary modifications are involved in intelligence (*P* = 1.7 × 10^−6^) and cortical area (*P* = 3.5 × 10^−4^) and exhibit elevated expression in language-related areas (*P* = 7.1 × 10^−4^), a hallmark of human cognition. Our findings suggest that recently evolved genetic variants shaped the human brain, cognition, and psychiatric traits.

## Introduction

Human evolution involved large-scale changes in brain structure and cognitive abilities (De Sousa et al. 2023). The human brain has tripled in volume since diverging from the last common ancestor with chimpanzees 5 to 6 million years ago (Mya; Rilling 2014). This growth accelerated over the past 2 million years, with exponential increases in cranial capacity among hominins, marking an exceptional rate of encephalization among mammals (Jerison 2012; Gómez-Robles et al. 2023). The neocortex underwent one of the most significant expansions (Stephan et al. 1988; Barton 2007a), prompting investigation into its role in the emergence of complex behavior and advanced cognition in the human lineage (Barton 2007b; Shultz et al. 2012; Wei et al. 2019; De Sousa et al. 2023).

Paleontological studies of archaic human skull endocasts provide key insights into changes in brain size and structure but offer limited insight into the evolution of brain function, cognition, and behavior, as these aspects do not fossilize (De Sousa et al. 2023). Our genome however preserves a footprint of human evolutionary history beyond physical records. Hominin encephalization likely resulted from the interplay of natural (e.g. climate), nutritional (e.g. diet), and social (e.g. group size, parental care) selection pressures (Sherwood et al. 2008), gradually shaping the human genome and driving adaptations that supported brain expansion and advanced cognition (Preuss et al. 2004). Investigating genomic changes over time can reveal traits subjected to evolutionary selection. Advances in genome-wide association studies (GWAS) have begun to unravel the genetic basis of modern human traits (Watanabe et al. 2019), offering new opportunities to trace the evolutionary timeline of human phenotypes.

In this study, we examined the temporal emergence of genetic variants related to brain and cognitive phenotypes by integrating genomic dating methods (Albers and McVean 2020) with GWAS data (Watanabe et al. 2019). We show that genetic variants associated with brain anatomy, cognitive abilities, and psychiatric disorders follow a distinctive temporal pattern, with these phenotypes undergoing the most recent genetic modifications in hominin evolution.

## Materials and methods

### Human genome dating

The Human Genome Dating (HGD) database (Albers and McVean 2020) was used to determine the emergence period of genetic variants in the human genome (source: https://human.genome.dating/). HGD infers the time of the most recent common ancestor between individual human genomes using recombination and mutation clocks (Albers and McVean 2020). Age estimates are provided for 13,689,983 single nucleotide polymorphisms (SNPs) across 22 chromosomes, based on sequencing data from the 1000 Genomes Project (The 1000 Genomes Project Consortium et al. 2015) and the Simons Genome Diversity Project (Mallick et al. 2016). These variants span ~ 200,000 generations (1 generation = 25 years), with estimated ages ranging from 5,140,625 to 87.5 years ago (75th–25th percentile: 659,445 to 39,735 years ago). The genomic dating method does not assume specific demographic or selective processes shaping the underlying genealogy (Albers and McVean 2020). This study used the median SNP age estimates derived from both clocks, combined across the 2 sequencing datasets. The HGD database assigns a quality score to each SNP, ranging from 0 (low) to 1 (high), reflecting confidence in the age estimate and allowing sensitivity analyses that prioritize the most reliable variants (Supplementary material).

### Phenotype-associated SNPs

We obtained genetic variants associated with human phenotypes from the GWAS Atlas (https://atlas.ctglab.nl, access date June 2021; Watanabe et al. 2019) by selecting lead SNPs representing associated genomic regions. Lead SNPs were identified using a 2-step clumping procedure: genome-wide significant SNPs (*P* < 5 × 10^−8^) were first clumped at R^2^ < 0.6, followed by a second clumping step at R^2^ < 0.1 to ensure independence within each GWAS. These SNPs were matched to the HGD SNPs ID (GRCh37) (Yates et al. 2016), and minor allele frequency (MAF) estimates were calculated using the Haplotype Reference Consortium panel (GRCh37) (the Haplotype Reference Consortium 2016).

A total of 33,621 unique SNPs with dating estimates were identified across 2500 GWAS, with their HGD time spanning 4,556,425 to 1681 years ago, referred to as phenotype-associated SNPs throughout this study. Phenotypes were hierarchically organized into domains (*n* = 24), chapters (*n* = 31), subchapters (*n* = 75), and trait levels (*n* = 361) (Watanabe et al. 2019). Analyses were restricted to phenotypes with at least 25 SNPs. GWAS examining related phenotypes were merged at the domain, chapter, and subchapter levels, while identical phenotypes were combined at the trait level (see Table S1 for a complete list of phenotypes).

Different GWAS on the same trait may identify distinct lead SNPs within overlapping Linkage Disequilibrium (LD) blocks, potentially biasing evolutionary age estimates. To address this, we applied the following filtering procedures simultaneously: (i) we selected independent SNPs within LD-blocks (R^2^ < 0.1; Slatkin 2008) and excluded those within the major histocompatibility complex (MHC) to ensure complete SNP independence across phenotypes (alternatively, SNPs in LD with any other SNPs within the same phenotype, regardless of R^2^, were excluded to maintain SNP independence within phenotypes); (ii) we analyzed only GWAS with sample sizes over 50,000 to improve statistical power and reliability of detected genetic associations, and (iii) we included only SNPs with a quality score above 0.7 to ensure accurate dating estimates. Findings were validated using independent data from the EBI GWAS Catalog (Buniello et al. 2019), incorporating 465 additional GWAS not included in the GWAS Atlas (*n* = 64,447 unique SNPs).

### Identification of evolutionary peaks in phenotype-associated SNPs

Peaks in the distribution of normalized SNP density over time were identified using 33,621 phenotype-associated SNPs from the GWAS Atlas. Peaks were defined as time points (across 100 intervals) where both neighboring points showed a smaller increase in SNP count (identified using Python package *SciPy* [Virtanen et al. 2020]). The 2 most prominent peaks were selected based on their vertical distance from the lowest contour line. An “old peak” ranged from 2,953,652 to 305,226 years ago, with a maximum at 1,104,751 years ago, while a “young peak” ranged from 305,226 to 1681 years ago, peaking at 54,119 years ago. Similar peak estimates were obtained when restricting the analysis to LD-independent phenotype-associated SNPs (R^2^ < 0.1) and variants outside the MHC.

### Genes evolutionary age estimation

Gene evolutionary age, complementary to SNP-based age, was estimated using a location-based approach. The median age of all SNPs within the transcription region of each gene (GRCh37) served as a proxy for gene age, assuming SNP distribution reflected the gene’s evolutionary history (see Supplementary material for details). This approach focused on genetic variation within the human lineage, complementing traditional phylogenetic methods that estimate gene age through cross-species sequence alignments (Kapli et al. 2020).

Evolutionary age estimates were obtained for 18,328 genes (median of 100 SNPs per gene), ranging from 2,965,600 to 3803 years ago (see Table S8 for complete list of genes ages). SNPs mapping to multiple genes or intergenic regions were excluded. Gene length showed a weak negative correlation with median evolutionary age (*r* = −0.058, *P* = 2.3 × 10^−15^), but controlling for gene length did not change the results (Supplementary material).

While we employed a location-based strategy, we acknowledge that distant SNPs can influence gene function (Uffelmann and Posthuma 2021), and future work integrating regulatory elements could refine these estimates. Sensitivity analyses incorporating a 1-kb window around each gene and using alternative genome assemblies (GRCh38 and CHM13) yielded similar results (Supplementary material).

### Statistical procedures

#### Temporal analysis

Non-parametric permutation testing was used to determine whether specific evolutionary periods showed an unusually high (or low) emergence of SNPs linked to human traits compared to chance. The genetic timeline of phenotype-associated SNPs was divided into 100 bins of 20,000-year intervals, with SNP counts recorded for each time bin. MAF matching was applied to account for differences in MAF distributions between phenotype-associated SNPs and all SNPs in the full HGD dataset. Specifically, SNPs from the total HGD pool were randomly sampled to match the MAF distribution of phenotype-associated SNPs across MAF bins (MAF ranges: < 0.00001, 0.00001–0.0001, 0.0001–0.001, 0.001–0.01, 0.01–0.1, 0.1–0.2, 0.2–0.3, 0.3–0.4, 0.4–0.5), yielding a MAF-matched pool of ~ 3.2 million SNPs. Next, a permutation null model was generated by randomly selecting 10,000 SNP sets from the MAF-matched pool, each matching the number of phenotype-associated SNPs. For each iteration, SNP counts within each time bin were recorded to generate a null distribution, which was used to compute *z*-scores and *P* values. Significant periods of SNP excess or depletion were identified using strict Bonferroni correction (*P* < 5 × 10^−4^) across the 100 bins. This analysis was restricted to SNPs younger than 2 million years, as they comprised 99.5% of phenotype-associated SNPs.

#### Evolutionary age of human phenotypes

Permutation testing was also used to evaluate whether the evolutionary age of genetic variants associated with specific phenotypes was significantly younger or older than expected by chance. A null model was generated by computing the median evolutionary age of randomly selected SNP or gene sets, each matched in size to the observed set. The relationship between MAF and variant age (r = 0.38, *P* < 1 × 10^−100^) was accounted for by drawing random SNPs from the total pool while preserving the MAF distribution of the observed set across MAF bins (MAF ranges: 0–0.1, 0.1–0.2, 0.2–0.3, 0.3–0.4, 0.4–0.5). This procedure was repeated 10,000 times to generate a null distribution of median evolutionary ages under the assumption of no association. The observed median evolutionary age of a phenotype was then compared to this null distribution to compute a *z*-score and *P* value, with a Bonferroni correction applied for multiple testing across phenotypes.

#### Gene-set analysis

MAGMA competitive gene-set analysis (De Leeuw et al. 2015) was conducted on the youngest (*n* = 1833; 53,077 to 3803 years ago) and oldest (*n* = 1833; 2,965,600 to 364,120 years ago) 10% of genes. Sensitivity analyses were performed using alternative gene thresholds (top 20%, 5%, and 1%) and accounted for gene length (see Supplementary material). Gene-based enrichment was examined for 5 neuropsychiatric disorders (schizophrenia [SCZ], bipolar disorder [BD], Alzheimer’s disease [AD], major depressive disorder [MDD], and autism spectrum disorder [ASD]), 3 brain-related phenotypes (brain volume [BV], cortical thickness [CT], and cortical area [CA]), and 2 cognitive traits (intelligence and sociability [SOC]; details in Table S9 and Supplementary material). Enrichment was tested separately in young and old gene sets (20 comparisons, Bonferroni *P* < 2.5 × 10^−3^). Gene-set analyses controlled for gene size, LD structure, mean minor allele count, and their log-transformed values.

#### Gene expression analysis

Permutation testing assessed the median expression of the youngest 10% of genes (*n* = 1635 out of 16,344 genes with expression and dating estimates, spanning 54,656 to 6717 years ago) using cortical gene microarray transcriptome data from the Allen Human Brain Atlas (Hawrylycz et al. 2012). Preprocessing details are provided in Supplementary material and (Wei et al. 2022). Sensitivity analyses tested alternative gene thresholds (top 20%, 5%, and 1%), gene age estimates, and brain parcellations (Supplementary material). GAMBA null-random-gene permutation testing (Wei et al. 2022) involved randomly selecting 10,000 gene sets of the same size as the observed set and estimating the median expression for each brain region. The observed median expression was compared to the null distribution to compute *z*-scores and *P* values, using a Bonferroni threshold to account for multiple comparisons across regions.

## Results

### Accelerated emergence in recent evolution of genetic variants linked to human phenotypes

The evolutionary timeline of SNPs associated with human phenotypic variation was reconstructed by integrating data from the HGD (Albers and McVean 2020) with summary statistics from 2500 GWAS (Watanabe et al. 2019) covering a broad range of phenotypes, including for example eye shape, cancer, height, brain anatomy, and aspects related to cognition and behavior.

**Fig. 1:**
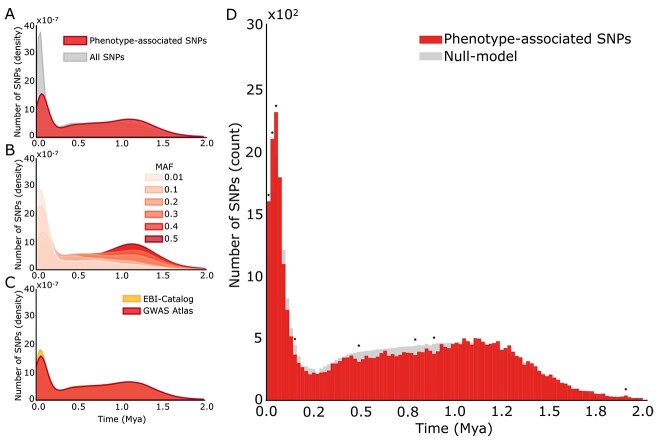
Genetic timeline of SNPs associated with human phenotypic variation. A) Density timeline (normalized count; *y*-axis) of phenotype-associated SNPs from the GWAS atlas (2500 GWAS, 36,506 unique SNPs) and all SNPs from the HGD over the last 2 million years (*x*-axis). HGD SNPs with MAF equal to or above the lowest MAF in phenotype-associated SNPs are shown (MAF ≥ 0.001; ~ 7.2 million SNPs). B) Density timeline of phenotype-associated SNPs grouped by MAF bins (0–0.01, 0.01–0.1, 0.1–0.2, 0.2–0.3, 0.3–0.4, 0.4–0.5). C) Validation analysis comparing the GWAS atlas phenotype-associated SNPs timeline to an alternative dataset from the EBI Catalog, showing high concordance (*rho* = 0.98, *P* = 2.8 × 10^−72^). D) Absolute SNP count per time bin (100 bins of ~ 20,000 years) for phenotype-associated SNPs and randomly selected SNPs. Asterisks denote bins where the number of phenotype-associated SNPs deviated significantly from the null model (MAF-controlled, 100 tests, Bonferroni *P* < 5 × 10^−4^). MAF, minor allele frequency.

A total of 33,621 genetic variants (referred to as phenotype-associated SNPs) were mapped from ~ 4.5 million to ~ 2000 years ago, showing 2 distinct periods of emergence (Fig. 1A). An older period (“old peak”) spanned ~ 2.9 million to ~ 300,000 years ago, peaking at ~ 1.1 M years ago, and contained ~ 85% of variants with high minor allele frequency (MAF ≥ 0.4; Supplementary material). This distribution suggests that variants shared by most modern humans emerged before the appearance of *Homo sapiens* (~300,000 to ~ 200,000 years ago) and the subsequent divergence of ancestral human populations (Vigilant et al. 1991; Hublin et al. 2017). The ancient origin of these common variants is consistent with their high frequency across geographically diverse human populations today (The 1000 Genomes Project Consortium et al. 2015). A recent period (“young peak”) occurred between ~ 300,000 and ~2000 years ago, peaking at ~ 55,000 years ago, and comprised ~ 60% of variants with low MAF (MAF ≤ 0.1; Fig. 1B). This allele frequency pattern reflects the time required for variants to increase in frequency through genetic drift or positive selection (Lynch et al. 2016). Validation using an independent set of 88,551 SNPs from the EBI Catalog of GWAS results (Buniello et al. 2019), which did not overlap with the phenotype-associated SNPs extracted from the GWAS Atlas, replicated the observed overall distribution (*rho* = 0.98, *P* = 2.8 × 10^−72^; Fig. 1C, Supplementary material).

Permutation testing compared the timeline of phenotype-associated SNPs against all SNPs to identify periods with a significantly higher or lower number of variants than expected (MAF-controlled, 10,000 permutations; Materials and Methods). A sharp increase in phenotype-associated SNPs occurred during the young peak (~60,000 to ~ 2000 years ago), with 3 consecutive significant time bins (*P* = 4.2 × 10^−16^, *P* = 6.2 × 10^−27^, and *P* = 2.3 × 10^−40^; Fig. 1D), indicating an accelerated emergence of genetic variants associated with human phenotypes in recent evolution. A milder increase was also detected at the onset of the old peak (~1,920,000 to 1,900,000 years ago, *P* = 2.5 × 10^−8^). Three periods showed significantly fewer phenotype-associated SNPs than expected: ~ 900,000 to ~ 880,000 years ago (*P* = 1.7 × 10^−4^), ~ 800,000 to ~ 780,000 years ago (*P* = 3.1 × 10^−4^), ~ 500,000 to ~ 480,000 years ago (*P* = 5.0 × 10^−5^), and ~ 160,000 to ~ 140,000 years ago (*P* = 4.2 × 10^−4^; Fig. 1D).

### Cognitive and psychiatric phenotypes are shaped by recent genetic modifications

Our analyses leveraged the hierarchical framework of the GWAS Atlas (Watanabe et al. 2019) to categorize the vast array of human traits and examine the emergence of genetic variants associated with these traits throughout evolution. This system organized 2500 GWAS into a nested taxonomy (Table S1), where broad “domains” (e.g. cognitive) contained more specific “chapters” (e.g. mental functions), which further divided into “subchapters” (e.g. memory) and individual “traits” (e.g. fluid intelligence) (Watanabe et al. 2019). This structure allowed the analysis of genetic timelines at multiple levels of phenotypic resolution, observing that SNPs underlying human phenotypes emerged progressively throughout evolution.

**Fig. 2:**
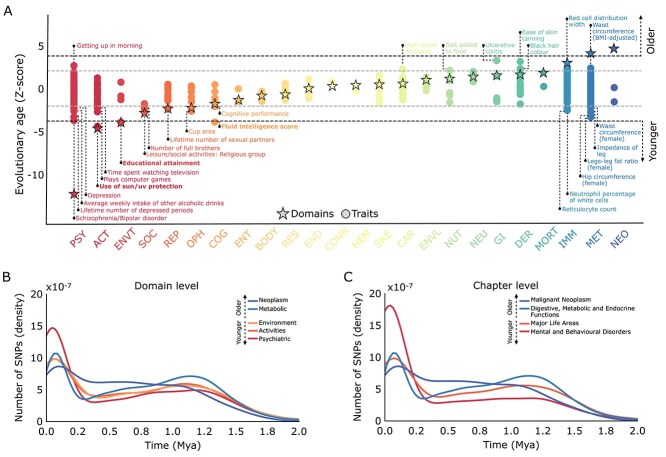
Genetic timeline of human traits. A) Genetic variants associated with human phenotypic variation were extracted from the GWAS atlas, which organizes phenotypes hierarchically into domains, chapters, subchapters, and trait levels. Expected trait ages (dots; *n* = 361) and domain ages (stars; *n* = 24) were estimated under a null model (see *evolutionary age of human phenotypes* in Materials and methods). *Z*-scores beyond the dotted black line indicate traits with a median age of SNPs significantly younger (negative *z*-scores) or older (positive *z*-scores) than expected by chance (controlling for polygenicity and MAF; Bonferroni *P* < 1.4 × 10^−4^). Gray dotted lines indicate nominal significance thresholds. Labels of nominally significant traits are displayed, with bold labels indicating Bonferroni significant effect. Colors denote each domain. B) Timeline of SNPs linked to human phenotypes at the domain level. Histogram showing the density (*y*-axis) of SNPs emerging over time (shown up to 2 Mya; *x*-axis). Domains with a significantly younger median age include “psychiatric”, “activities”, and “environment”, while “metabolic” and “neoplasms” are significantly older (controlling for polygenicity and MAF; *n* = 24 domains, Bonferroni *P* < 2.1 × 10^−3^). C) Timeline of SNPs linked to human phenotypes at the chapter level. Histogram showing the density (*y*-axis) of SNPs emerging over time (shown up to 2 Mya; *x*-axis). Chapters with a significantly younger median age are “mental and behavioral disorders” and “major life areas”, while “digestive, metabolic and endocrine systems” and “malignant neoplasms” are significantly older (controlling for polygenicity and MAF; *n* = 31 chapters, Bonferroni *P* < 1.6 × 10^−3^). ACT, activities; BMI, body mass index; BODY, body structures; CAR, cardiovascular; CONN, connective tissue; DER, dermatological; END, endocrine; ENVT, environment; ENVL, environmental; GI, gastrointestinal; HEM, hematological; IMM, immunological; MET, metabolic; MORT, mortality; NEO, neoplasms; NEU, neurological; NUT, nutritional; OPH, ophthalmological; PSY, psychiatric; REP, reproduction; RES, respiratory; SKE, skeletal; SOC, social interactions.

Statistical testing across all 24 domains assessed whether genetic variants linked to specific phenotypes were significantly younger or older than expected based on polygenicity and allele frequency. While variants associated with “Psychiatric” phenotypes emerged across a broad period of time between ~ 3.5 million to ~ 3000 years ago, collectively they had a significantly younger median age than average (475,833 years; *P* = 5.9 × 10^−33^; Fig. 2A and B). Similarly, variants linked to “Activities” (e.g. medication use, physical activity levels; 644,450 years; *P* = 5.2 × 10^−6^) and “Environment” phenotypes (e.g. educational attainment, perinatal and socioeconomic health risks factors; 626,965 years; *P* = 1.1 × 10^−4^) were also significantly younger. In contrast, variants associated with “Neoplasm” (591,690 years; *P* = 1.1 × 10^−5^) and “Metabolic” phenotypes (785,572 years; *P* = 6.6 × 10^−5^) had older median ages (Fig. 2A and B; all *P* values in Table S2).

Findings were consistent across chapters (Fig. 2C), subchapters (Fig. S1), and traits (Fig. S2), with cognitive and psychiatric phenotypes repeatedly showing younger evolutionary ages (Table S2). In particular, educational attainment (*P* = 1.3 × 10^−4^), fluid intelligence (*P* = 1.4 × 10^−4^), depression (*P* = 1.6 × 10^−4^), and alcoholism-related traits (*P* = 5.2 × 10^−12^) were among the youngest phenotypes. To expand the coverage of examined GWAS, the EBI Catalog (Buniello et al. 2019) was incorporated, adding 465 studies not included in the GWAS Atlas. Similarly, variants linked to cognition (“General cognitive ability”, *P* = 5.9 × 10^−5^; “Cognitive aspects of educational attainment”, *P* = 9.1 × 10^−5^) and mental health (“Deliberate self-harm”, *P* = 3.2 × 10^−5^) were also among the youngest phenotypes (Supplementary material; Table S3). Additional sensitivity analyses (Supplementary material) confirmed the main findings by applying 3 simultaneous filters: (i) removing SNPs in LD (R^2^ > 0.1) to ensure independence across phenotypes (or alternatively, removing SNPs in LD within the same phenotype); (ii) accounting for GWAS sample size differences; and (iii) controlling for variant dating accuracy.

We validate our findings with an alternative allele age data [ARGweaver, (Rasmussen et al. 2014)] using GSEL (Abraham et al. 2023), a comprehensive statistical method for assessing trait-level enrichment of genetic variants under evolutionary selection. GSEL tested SNP ages against a null distribution of MAF- and LD-matched variants (Supplementary material), confirming that variants associated with intelligence (*P* = 1 × 10^−4^), SCZ (*P* = 4.2 × 10^−3^), and MDD (*P* = 0.040) display significantly younger evolutionary ages (for complete results, see Table S4).

### Recent evolutionary genetic variants contribute to cortical variation

After confirming that genetic variants associated with cognitive and psychiatric traits emerged recently in human evolution, we systematically investigated the evolutionary timelines of genetic variants linked to brain structures, with a particular focus on the cortex, which underwent extensive changes in hominins (Barton 2007a). This analysis included 2273 variants (BRAIN-SNPs) collated from extensive GWAS of 1138 neuroimaging-derived phenotypes in the UK Biobank (Smith et al. 2021), categorized by their association with specific brain structures (see Table S5). The timeline of BRAIN-SNPs spanned ~ 3.6 million to ~ 5000 years ago, with a median age of 735,652 years (Fig. S4).

Statistical analysis controlling for polygenicity and MAF distribution indicated that cortex-related variants (*n* = 126 SNPs, 400,170 years) showed a borderline trend toward younger age compared to other BRAIN-SNPs, though the effect was not statistically significant (*P* = 0.06; Fig. S5a, Table S6; Supplementary material). Additional analyses using ENIGMA GWAS summary statistics on cortical and subcortical structures provided stronger evidence, revealing that variants associated with CA (*n* = 827 SNPs, 289,780 years) and CT (*n* = 296 SNPs, 279,068 years) were significantly younger than average (*P* = 9.1 × 10^−72^ and 4 × 10^−28^, respectively; Supplementary material). Further validation with GSEL confirmed that genetic variants linked to CA (*P* = 1.2 × 10^−3^) and BV (*P* = 4.4 × 10^−3^; Table S4) displayed younger evolutionary ages.

### Evolutionary age of human genes

**Fig. 3:**
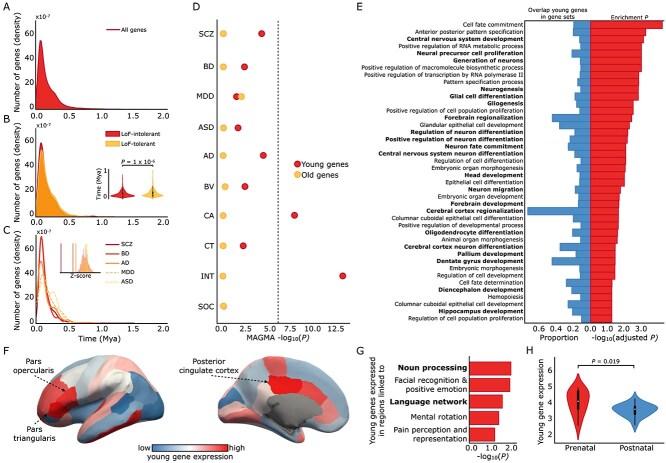
Enrichment analysis and transcriptomic brain map of evolutionarily recent genes. A) Evolutionary timeline of genes (*n* = 18,328) with age estimates ranging from 2,965,600 to 3803 years ago. The *y*-axis represents density distribution, and the *x*-axis shows gene age bins (only genes younger than 2 million years are displayed). B) Evolutionary timeline of genes ranked by intolerance to LoF mutations. The *y*-axis shows the median evolutionary age of the most LoF-intolerant genes and LoF-tolerant genes. LoF-intolerant genes are significantly younger (*t*-test, *P* = 8.1 × 10^−6^). C) Evolutionary timeline of genes associated with 5 major neuropsychiatric disorders. Solid lines indicate phenotypes significantly younger than expected under the null model (Bonferroni *P* < 5 × 10^−3^) for SCZ, BD, and AD. The insert panel shows null distributions for each condition. D) MAGMA gene-set analysis testing for enrichment of genes related to brain, cognition, and neuropsychiatric among the oldest and youngest genes. The *x*-axis shows *P* values, while the *y*-axis lists tested phenotypes. The dotted line indicates the Bonferroni significance threshold (*P* < 2.5 × 10^−3^). Young genes show strong enrichment for intelligence and CA, and nominal associations for AD and SCZ. No enrichment is observed for brain-related traits or neuropsychiatric disorders in old genes. E) Functional annotation of young genes identified 21 out of 42 significant biological functions related to the brain (*q* < 0.05, FDR). Bold text indicates significant enrichment for brain-related biological processes. F) Normative expression levels of the young genes across brain areas, ranging from relatively low to high expression levels (*z*-scores). The figure shows higher normalized expression in Broca’s area (pars triangularis: *Z* = 3.4, *P* = 7.1 × 10^−4^; pars opercularis: *Z* = 2.6, *P* = 8.5 × 10^−3^), a critical region for language processing, as well as in the posterior cingulate (*z* = 2.7, *P* = 7.0 × 10^−3^), a key hub of the default mode network. G) Young genes show significant overexpression (*P* < 0.05) in regions linked with 5 cognitive domains (*n* = 111 total terms) from the Neurosynth database (meta-analysis of over 10,000 functional brain studies). Cognitive domains related to language are highlighted in bold. H) Young genes exhibit significantly higher expression in prenatal stages (8 to 37 weeks post-conception) compared to postnatal stages (4 months to 40 years old). AD, Alzheimer’s disease; ASD, autism spectrum disorder; BD, bipolar disorder; BV, brain volume; CA, cortical area; CT, cortical thickness; INT, intelligence; LoF, Loss-of-Function; MDD, major depressive disorder; Mya, million years ago; SCZ, schizophrenia; SOC, sociability.

Analyses of genetic variants uncovered evolutionary patterns in human phenotypes, and examining these variants within genes may provide further insights into their biological significance. The median evolutionary age of variants within each gene’s transcription region was calculated, providing age estimates for 18,328 human genes (see Table S8 for gene age estimates). Gene ages ranged from ~ 3 million to ~ 4000 years ago, peaking at ~ 70,000 years ago (Fig. 3A).

We examined the relationship between mutation resilience and evolutionary age by ranking genes based on their intolerance to loss-of-function (LoF) mutations (Supplementary material) (Balasubramanian et al. 2017). As expected, the most LoF-intolerant genes (top 10%) were significantly younger than LoF-tolerant genes (*t* = −4.47, *P* = 8.1 × 10^−6^; Fig. 3B), indicating that genes highly intolerant to functional disruption tend to contain evolutionarily younger variants. These findings were confirmed through sensitivity analyses (see Supplementary material).

### Recent evolution of genes associated with brain, cognition, and neuropsychiatric disorders

We tested whether genes associated with brain, cognition, and neuropsychiatric disorders (identified through gene analysis of GWAS summary statistics; *q* > 0.05, FDR; Supplementary material) had younger evolutionary ages compared to other human genes (Bonferroni *P* < 5 × 10^−3^). Genes linked to intelligence (*n* = 2102 genes; *P* = 6.4 × 10^−17^), SCZ (*n* = 2275 genes; *P* = 8.1 × 10^−10^), BD (*n* = 357 genes; *P* = 5.5 × 10^−5^), SOC (*n* = 250 genes; *P* = 1.4 × 10^−4^), BV (*n* = 170 genes; *P* = 1.6 × 10^−4^), and AD (143 genes; *P* = 2.6 × 10^−3^) were significantly younger than other human genes (Figs. 3C and S6; sensitivity analysis in Supplementary material).

### Evolutionarily recent genetic modifications underlying human cortex and cognition

We focused on the most recently modified genes during evolution and examined their functional significance. Gene-set analysis using MAGMA (controlling for LD structure, gene size, and mean minor allele count) (De Leeuw et al. 2015) of the youngest 10% genes (~53,000 to ~ 4000 years ago) revealed significant enrichment for intelligence (*P* = 1.7 × 10^−6^) and CA (*P* = 3.5 × 10^−4^). Young genes also showed nominal associations with AD (*P* = 0.011) and SCZ (*P* = 0.014; Fig. 3D), though these effects did not survive strict Bonferroni correction for 20 tests (full results in Table S10). Sensitivity analyses varying gene inclusion thresholds and controlling for gene length confirmed these findings (Supplementary material). This enrichment was specific to young genes, as the oldest genes showed no enrichment for brain, cognitive, or psychiatric phenotypes (~3 million to ~ 360,000 years ago; *P* > 0.05; Fig. 3D, Table S10).

Functional annotation using FUMA (Supplementary material) (Watanabe et al. 2017) of young genes revealed enrichment for 42 biological functions (Fig. 3E), 21 of which were related to the brain, including neurogenesis (*P* = 1.2 × 10^−6^), cortical neuron differentiation (*P* = 1.9 × 10^−4^), and the development of the forebrain (*P* = 5.2 × 10^−5^), diencephalon (*P* = 2.2 × 10^−4^), and hippocampus (*P* = 2.3 × 10^−6^).

### Expression of young genes in language-related brain areas

We investigated whether genes containing recently evolved variants are preferentially expressed in specific cortical regions by analyzing microarray data from the Allen Human Brain Atlas (Hawrylycz et al. 2012). Young genes (top 10%, ~ 54,000 to ~ 7000 years ago) exhibited significantly higher expression in the pars triangularis (*P* = 7.1 × 10^−4^; Fig. 3F, Table S11) and nominally higher expression in the pars opercularis (*P* = 8.5 × 10^−3^), both key regions of Broca’s area, central to language processing (Friederici 2011). Indeed, young genes showed significant overexpression in language-related regions [identified through a meta-analysis of brain functional imaging studies from the Neurosynth database (Yarkoni et al. 2011), see Supplementary material and Fig. 3G], with no comparable effects observed in other evolutionary time bins (*P* > 0.05). Young gene expression was also nominally higher in the posterior cingulate cortex (*P* = 7.0 × 10^−3^; Fig. 3F), a default mode network hub involved in self-referential thinking and memory retrieval (Raichle 2015), and in the inferior temporal gyrus (*P* = 9.0 × 10^−3^), critical for visual object recognition and memory encoding (Conway 2018). In contrast, expression was significantly lower in the lateral occipital gyrus (*P* = 1.1 × 10^−3^), a region involved in visual processing (Grill-Spector et al. 2001). Sensitivity analyses using different gene thresholds, cortical parcellations, and alternative gene age estimates yielded consistent results (Supplementary material). Further analysis of the BrainSpan Atlas of the Developing Human Brain (Miller et al. 2014) (Supplementary material) showed that genes with recent modifications were more highly expressed during prenatal compared to postnatal stages (*P* = 0.019; Figs. 3H and S7), while the oldest genes showed no significant difference between these stages (*P* > 0.05).

## Discussion

Examining the timeline of genetic modifications in the human genome revealed distinct evolutionary trajectories for human phenotypic variants. Variants linked to brain surface and volume, intelligence, and psychiatric disorders emerged more recently than those associated with other phenotypes. These findings align with evidence of ongoing cortical remodeling and cognitive specialization throughout hominin evolution (Preuss et al. 2004; De Sousa et al. 2023).

Our analyses identified 2 major periods of variant emergence during hominin evolution: an “old peak” (~2.9 million to ~ 300,000 years ago) and a “young peak” (~300,000 to ~ 2000 years ago). These peaks correspond with key paleontological milestones and align with comparative genomic analyses which also identify 2 major adaptive shifts in the human genome ~ 600,000 and ~ 200,000 years ago (Schaefer et al. 2021).

The old peak coincides with the emergence of *Homo Habilis* (~2.8 million to ~ 2 Mya) (Spoor et al. 2015; Villmoare et al. 2015; Antón and Middleton 2023) and the later appearance of *Homo erectus* (~1.9 million to ~ 1.5 Mya) (Herries et al. 2020; Antón and Middleton 2023). This period is known for significant evolutionary developments in hominins, including the origins of *Homo Heidelbergensis* (~700,000 years ago) (Stringer 2012; Buck and Stringer 2014) and the divergence of *H. sapiens* and Neanderthals (~800,000 to ~ 600,000 years ago) (Prüfer et al. 2014; Gómez-Robles 2019), as well as notable increases in brain size (Coqueugniot et al. 2004; Leigh 2012), bipedal skeletal adaptations (Antón 2003; Hatala et al. 2016), and early fire use (~1.5 million to ~ 1 Mya) (Berna et al. 2012; Hlubik et al. 2017).

The young peak in turn aligns with the origin of *H. sapiens* (~300,000 to ~ 200,000 years ago) (Vigilant et al. 1991; Hublin et al. 2017) and the dispersal of *H. sapiens* out of Africa (~60,000 years ago) (Posth et al. 2016). Our analyses show a surge in phenotype-associated SNPs over the last 60,000 years (Fig. 1D), consistent with evidence of an accelerated emergence of modern human-specific variants ~ 40,000 years ago (Andirkó et al. 2022). This timing coincides with environmental adaptations during human migration from Africa to Eurasia (~70,000 to ~ 40,000 years ago) (Soares et al. 2012; Posth et al. 2016) and the Upper Paleolithic (~45,000 years ago) (Bar-Yosef 2002). This last period marks the earliest consistent evidence of symbolic behavior (e.g. art carvings), technological innovation (e.g. microlithic stone tools), and ecological expansion (e.g. long-distance trading) in humans (d’Errico et al. 2003). While the observed surge in genetic variants aligns with Upper Paleolithic transitions, the timing of cognitive and cultural evolution remains debated, with evidence suggesting complex cognitive behavior in hominins as early as ~ 300,000 years ago (Mcbrearty and Brooks 2000; Henshilwood et al. 2001; Brooks et al. 2018).

Genetic modifications have played a crucial role in brain growth and remodeling (Preuss et al. 2004), driving substantial changes in size and structure since the last common ancestor of modern humans and other primates (Finlay and Darlington 1995; Van Essen et al. 2018; Ardesch et al. 2019). The cortex is one of the most expanded brain structures (Stephan et al. 1988; Barton 2007b), and its size sets humans apart from other primates (Barton 2007b; Shultz et al. 2012; Wei et al. 2019). This expansion is believed to be crucial for advanced cognitive functions, including complex language, social cognition, and higher-order thinking (Ardesch et al. 2019; Changeux et al. 2021; De Sousa et al. 2023). Our findings suggest that genetic modifications related to cortical morphology and intelligence are among the most recent in human evolution (Fig. 3C and D), reflecting ongoing genetic influences on brain structure and cognitive abilities. It is important to note that the genetic variants examined here are linked to interindividual differences in brain size, rather than reflecting evolutionary trends in brain expansion. Brain size has fluctuated over time in humans (Holloway et al. 2009), with some theories even suggesting a decline over the past ~ 3000 years (DeSilva et al. 2021).

Human linguistic capacity is a distinctive feature that differentiates humans from other apes (Friederici 2017). The human cortex is particularly enlarged in language-related regions such as Brodmann areas 44 and 45 (Schenker et al. 2010), as well as an expanded arcuate fasciculus pathway interconnecting these language areas (Rilling et al. 2008; Friederici 2017). Gene expression patterns suggest that language-related brain regions underwent distinct evolutionary changes during recent human history (~54,000 to ~ 7000 years ago), with genes highly expressed in frontal areas, particularly in the gyrus pars triangularis, a key region in Broca’s area involved in language processing and speech production (Friederici 2017). This aligns with findings that alleles linked to the overall size of these language-related brain regions have undergone recent selection pressures (Tilot et al. 2021).

Human-accelerated regions (HAR) of the genome (Pollard et al. 2006) have been implicated in the neurobiological foundation of human brain and cognition. HAR genes are highly expressed in multimodal and language-related brain areas (Wei et al. 2019), influence social behavior (Doan et al. 2016), and contribute to human-specific neurodevelopmental brain rewiring (Girskis et al. 2021). HAR genes were found to display on average an older age than other human genes (*P* = 0.043; Supplementary material), while young variants linked to cortical organization are highly enriched within HAR genes (*P* = 1 × 10^−15^, Supplementary material). This supports an evolutionary model where HAR genes may have initially established critical neurodevelopmental scaffolding (Pollard et al. 2006) in early human evolution, with later-evolving variants potentially refining cortical structure by further modifying these genomic regions.

Genetic modifications have driven adaptations in brain structure and neural circuits, supporting the development of advanced cognitive skills (Bailey and Geary 2009; Shultz et al. 2012; Ardesch et al. 2022). These same changes may have also rendered the human brain vulnerable to dysfunction, as psychiatric and neurodegenerative disorders are highly prevalent in humans and occur at a scale not seen in other species (Crow 2000; Finch and Austad 2015; Doan et al. 2016; Van Den Heuvel et al. 2019; Pattabiraman et al. 2020). Our analysis supports this idea, revealing a progressive evolutionary timeline in which genetic variants broadly associated with the nervous system (~800,000 years) emerged before those linked to cognition (~680,000 years; 2-sided *t*-test, *t* = 2.70, *P* = 7 × 10^−3^), which in turn preceded variants associated with psychiatric disorders (~475,000 years; *t* = 7.29, *P* = 3.7 × 10^−13^; Fig. S3). These findings align with the hypothesis that brain reorganization and cognitive advancements in human evolution may have come at the cost of increased susceptibility to brain dysfunction (Van Den Heuvel et al. 2019; Pattabiraman et al. 2020).

The genetic variants related to psychiatric disorders showed some of the most recent evolutionary changes compared to other phenotypes. Depression (~24,000 years) and alcoholism-related phenotypes (~40,000 years; see subchapter level in Supplementary material) underwent particularly recent genetic modifications. These findings align with evidence linking introgressed Neanderthal variants (~60,000 to ~ 40,000 years ago) to smoking, alcohol consumption, and mood-related traits (Dannemann et al. 2022). Additionally, mood-related alleles are overrepresented in the genomes of ancient farmers (~11,000 years old) but not in earlier hunter-gatherers, who predate agriculture (Irving-Pease et al. 2024). Genes with recent modifications (within the last ~ 53,000 years ago) are also associated with cortical surface area and intelligence, aspects often implicated in psychiatric and neurological conditions (Thompson et al. 2020).

Our results support the notion that variants related to cognitive and psychiatric phenotypes have become more prevalent faster than other SNPs throughout evolution, possibly due to selection pressures (Pritchard et al. 2010; Karlsson et al. 2014; Simons et al. 2022). Genetic variants linked to psychiatric disorders may have been retained due to adaptive advantages in brain function (Pollard et al. 2006; Doan et al. 2016; Wei et al. 2019), immune response (Raison and Miller 2013; Lynall et al. 2022; Yang et al. 2023), or enhanced reproductive success (Escott-Price et al. 2019; Ni et al. 2019). Supporting the latter hypothesis, our analyses reveal that variants linked to sexual traits (e.g. lifetime number of sexual partners) have a younger evolutionary age than average (see Fig. 2A, Table S2), a trait that has also been implicated in psychiatric disorders such as depression (Lu et al. 2023).

This study focused on modern human genetic evolution, but its relationship to ancient hominins or other species remains unresolved. Independent evolutionary lineages facing similar ecological pressures can develop analogous neural circuits through shared molecular mechanisms. For instance, humans and songbirds exhibit convergent evolution in vocal-learning circuits, with overlapping transcriptomic signatures supporting complex vocalization (Pfenning et al. 2014). The genes underlying these molecular signatures [Table S6 from (Pfenning et al. 2014)] have an older evolutionary age than average (*P* = 4.8 × 10^−4^), suggesting that genetic modifications supporting human vocalization emerged early in hominin evolution.

In contrast, comparisons with closely related species highlight unique aspects of human cognition. Humans and chimpanzees share conserved features, including hemispheric asymmetry (Neubauer et al. 2020), macroscale brain networks (Li et al. 2013), and cortical circuits involved in problem-solving, relational reasoning, and sensory perception (Rilling 2014; van den Heuvel et al. 2023; Oyama et al. 2024). Neurobiological differences also exist between species (Rilling 2014), with humans displaying unique connectivity patterns critical for language, while chimpanzees exhibit superior working memory and auditory processing capabilities (Rilling et al. 2008; van den Heuvel et al. 2023). Future research integrating genomic dating data with cross-species genomic, neuroimaging, and single-cell transcriptomic data (Siletti et al. 2023) could further elucidate the genetic and molecular mechanisms shaping human cognitive traits.

Mapping the genetic timeline of human traits presents methodological challenges. Our findings should be interpreted with caution, as the majority of available GWAS data (~90%) focus on European populations (Mills and Rahal 2019), which may not fully capture the global diversity of human evolutionary history. The genomic data used in this study are based on SNP arrays, which can introduce genotyping errors in complex genomic regions, and limit the number of SNPs available for analysis. Future studies could mitigate these limitations by incorporating higher-quality sequencing technologies, such as long-read assemblies (Nurk et al. 2022; Wang et al. 2022). This study primarily examines common variants identified in GWAS, which may bias evolutionary age estimates by excluding rare and fixed variants. Consequently, median evolutionary age estimates of traits should be interpreted with caution, considering both lower and upper bounds (see Table S7). Additionally, comparisons across traits are challenging, as biological markers have a stronger genetic basis than behavioral phenotypes, influencing their polygenicity (Watanabe et al. 2019). To account for these factors, our null models control for polygenicity and MAF distribution, considering the diverse genetic architecture of human traits. Although this approach does not directly control for LD, sensitivity analyses restricted to independent SNPs yielded similar results (Supplementary material). Finally, aligning genetic timelines with human evolutionary milestones does not establish causality regarding the selective forces shaping the genome.

## Supplementary Material

Supplementary_Information_bhaf127

Supplementary_Table_1_bhaf127

Supplementary_Table_2_bhaf127

Supplementary_Table_3_bhaf127

Supplementary_Table_4_bhaf127

Supplementary_Table_5_bhaf127

Supplementary_Table_8_bhaf127

## Data Availability

HGD is available at https://human.genome.dating. GWAS Atlas is available at https://atlas.ctglab.nl. EBI Catalog is available at https://www.ebi.ac.uk/gwas. UK Biobank BIG40 is available at https://open.win.ox.ac.uk/ukbiobank/big40. Summary statistics of GWAS from brain disorders, intelligence, and brain volume are available at https://www.med.unc.edu/pgc/download-results; from cortical area and thickness are available at https://enigma.ini.usc.edu/research/download-enigma-gwas-results; and from sociability is available at https://www.repository.cam.ac.uk/handle/1810/277812. Cortical gene microarray transcriptome expression data from the Allen Human Brain Atlas is available at http://human.brain-map.org/static/download. Neurosynth database is available at https://neurosynth.org/.
