# Supplementary material for: The emergence of genetic variants linked to brain and cognitive traits in human evolution
Source: Cereb Cortex. 2025 Aug 13;35(8):bhaf127. doi: 10.1093/cercor/bhaf127 (PMC12345208; doi:10.1093/cercor/bhaf127)
Supplement: Supplementary_Information_bhaf127 [file supplementary_information_bhaf127.docx]

**Supplementary information for**

The emergence of genetic variants linked to brain and cognitive traits in human evolution

**Authors**

Ilan Libedinsky, Yongbin Wei, Christiaan de Leeuw, James K. Rilling, Danielle Posthuma, Martijn P. van den Heuvel

**Corresponding author:** Ilan Libedinsky

**E-mail:** i.libedinsky@vu.nl

**This file includes:**

Supplementary Methods and Supplementary Results

Supplementary Figure 1 to 7

Supplementary Tables 1 to 11 (Supplementary Table 1-5, 7 in separate files)

**Supplementary Methods**

**SNP-level analyses**

***MAF evolutionary age analysis.*** Minor allele frequencies (MAF) were calculated using the Haplotype Reference Consortium panel (the Haplotype Reference Consortium 2016) (GRCh37). Phenotype-associated SNPs MAF values were rounded and categorised into high MAF (MAF ≥ 0.4, n = 7,581 SNPs) and low MAF variants (MAF ≤ 0.1, n = 6,281 SNPs; Figure 1B). Permutation testing was used to assess whether the proportion of low or high MAF SNPs within old/young peaks was significantly different from expectation. By randomly selecting the same number of high/low MAF SNPs from the total pool of human phenotype-associated SNPs, the proportion of random SNPs falling within the old/young peak was estimated. This process was repeated 10,000 times, and a *z*-score with a corresponding *P* value was assigned based on the null distribution of random effects.

***EBI human-phenotypic timeline.*** The genetic timeline of phenotype-associated SNPs was also computed using GWAS results of the EBI Catalog (Buniello et al. 2019), which includes 4,238 GWAS detecting 88,551 non-duplicated SNPs. Only SNPs absent from the GWAS Atlas were selected to ensure that similarities between the GWAS Atlas and EBI Catalog distributions were not driven by overlapping SNPs (Figure 1C). The analysis included SNPs with an evolutionary age younger than 2 million years, based on the observation that 99.5% of human phenotypic-associated SNPs fall within this range. The timeline was divided into 100 bins of equal duration (~20,000 years), and SNP counts per bin were computed separately for each dataset. Spearman’s correlation was used to assess the similarity in the temporal distribution of emerging SNPs between the GWAS Atlas and EBI Catalog.

***LD correction.*** The estimation of phenotype ages from the GWAS Atlas was corrected for LD across all human phenotype-associated SNPs by including only independent SNPs within LD blocks, identified using PLINK (Purcell et al. 2007) (v1.9) with the pruning function --indep-pairwise (window size = 50, step size = 10, R^2^ < 0.1), on Phase 3 reference data from the 1,000 Genomes (The 1000 Genomes Project Consortium et al. 2015); European population). Additionally, SNPs within the MHC region (chromosome 6, base pairs between 28477797 - 33448354, GRCh37) were excluded. This procedure yielded 5,651 unique SNPs, with evolutionary ages ranging from 3,621,625 to 3,129 years ago.

**BRAIN-SNPs.** The extensive UK Biobank BIG40 GWAS (https://open.win.ox.ac.uk/ukbiobank/big40/) (Smith et al. 2021) identified 2,273 unique SNPs (with available MAF and dating estimates) significantly associated with 1,138 brain phenotypes (*P* < 5 x 10^−8^, discovery sample), with evolutionary ages ranging from 3,621,625 to 4,904 years ago. These BRAIN-SNPs were used to assess the evolutionary age of variants linked to specific brain structures. Variants were grouped into nine brain structures: cortex, cerebellum, hippocampus, amygdala, thalamus, nucleus accumbens, caudate nucleus, pallidum, and white matter (for a complete list of neuroimaging-derived phenotypes and their organisation within these brain structures, see Table S5). For validation, genome-wide significant SNPs (P < 5 x 10^-8^) from ENIGMA GWAS summary statistics were incorporated. These included subcortical structures (hippocampus, putamen, and amygdala; no significant SNPs were found for the other four available subcortical structures) (Hibar et al. 2015), cerebellum and overall subcortical structures (Tissink et al. 2022), as well as cortical surface and thickness (Grasby and Jahanshad 2020).

***BRAIN-SNPs timeline co-fluctuation.*** The temporal co-fluctuations of SNPs associated with brain structures were assessed by dividing the time dimension into 100 bins (~36,500 years each) and counting the number of SNPs appearing in each bin for each brain structure. Spearman's correlation was used to evaluate the similarity in genetic timelines across brain structures (36 tests, Bonferroni *P* value threshold < 1.4 x 10^-3^; Figure S5B). Overlapping SNPs between structures were excluded from the analysis. Due to the low frequency of SNPs predating 2 million years ago, the analysis was repeated using only SNPs younger than this threshold (see *BRAIN-SNPs timeline co-fluctuation validation* in Supplementary Results).

**Human accelerated regions.** We investigated whether SNPs associated with each brain structure overlap with genes located in the human accelerated regions (HARs) of the genome (Pollard et al. 2006). Out of 2,161 HAR genes (Doan et al. 2016), 1,722 had genomic position data (GRCh37). For each brain structure from BIG40 (Smith et al. 2021), the number of BRAIN-SNPs within HAR genes was calculated and compared this to a null distribution. This null distribution consisted of 10,000 randomly selected BRAIN-SNPs sets matched for size, evolutionary age (± 5,000 years), MAF (± 0.05) relative to the investigated neuroimaging-derived phenotype. A *P* value was computed as the proportion of null conditions in which the number of SNPs within HAR genes was greater than or equal to the observed count.

Additionally, we tested whether HAR genes have a younger or older median evolutionary age by randomly selecting gene sets of the same size (10,000 iterations) and computing a *z*-score and *P* value to compare the observed age of HAR genes against the null distribution.

**Enrichment for signatures of evolutionary forces.** We applied GSEL, a tool designed to detect evolutionary forces in GWAS summary statistics by comparing observed evolutionary measures to a null distribution of matched SNPs (Abraham et al. 2022, 2023). Briefly, independent genomic regions were identified by pruning based on LD (R^2^ ≥ 0.9), genomic distance (≤ 500 kilobases), and genome-wide significance (*P* < 5 x 10^-8^). For each lead SNP, 5,000 control SNPs were randomly selected to match minor allele frequency (±5%) and LD structure (number of SNPs with R^2^ ≥ 0.9). The number of variants in LD with a control SNP (R^2^ > 0.9) was matched to the number of SNPs in LD with the lead SNP. For each genomic region, the highest evolutionary value among matched SNPs was estimated and averaged across regions. *P* values were calculated by determining how often the trait’s evolutionary values met or exceeded those of the matched evolutionary measures. This analysis used an alternative resource for allele age estimation, ARGweaver (Rasmussen et al. 2014), instead of HGD.

**Gene-level analysis**

***Genes evolutionary age estimation.*** Gene evolutionary age was estimated by calculating the median age of SNPs within each gene’s transcription region (GRCh37). SNPs mapping to multiple genes or intergenic regions were excluded (58% of all SNPs). Median evolutionary age estimates were obtained for 18,328 genes (out of 26,836), each containing at least one SNP with a date estimate (median of 100 SNPs per gene), with ages ranging from 2,965,600 to 3,803 years ago (evolutionary ages for all genes are reported in Table S8). Gene length showed a weak negative correlation with median evolutionary age (*r* = -0.058, *P* = 2.3 x 10^-15^). The median evolutionary age was used as the primary metric for contextualizing gene ages within human evolution, and regressing out gene length confirmed that gene size did not influence the findings

***Sensitivity analysis using GRCh38 and CHM13 assemblies.*** SNPs from the HGD database were lifted over from GRCh37 to GRCh38 assemblies (using Python package *pyliftover*, based on UCSC liftOver genome coordinate conversion), and gene evolutionary ages were recalculated based on GRCh38 gene positions. Gene ages between GRCh37 and GRCh38 showed a strong correlation (Pearson’s r = 0.97), indicating highly similar estimations. Of the 18,066 genes, 71% retained the same age, while 29% differed, with a median absolute difference of 4,266 years (across a 5 million years timespan). Among genes with age differences, correlation remained high (Pearson’s r = 0.83). Permutation testing using median gene ages from GRCh38 produced highly similar results, confirming that genes linked to brain volume (*P* = 2 x 10^-4^), intelligence (*P* = 8.4 x 10^-18^), sociability (*P* = 1.1 x 10^-4^), schizophrenia (*P* = 3.6 x 10^-17^), and bipolar disorder (*P* = 9.1 x 10^-5^) had a younger evolutionary age than expected. Additionally, young genes showed higher transcriptomic expression in the pars triangularis (*P* = 9.6 x 10^-3^).

A similar sensitivity analysis was conducted using the CHM13 assembly from the Telomere-to-Telomere Consortium (Nurk et al. 2022). Gene ages between GRCh37 and CHM13 were highly correlated (Pearson’s r = 0.93). Of the 16,562 genes, 57% differed in age, with a median absolute difference of 4,607 years. Among genes with age differences, correlation remained high (Pearson’s r = 0.83). Permutation testing using CHM13 median gene ages confirmed that genes associated with brain volume (*P* = 3.5 x 10^-4^), intelligence (*P* = 1.5 x 10^-17^), sociability (*P* = 1.2 x 10^-4^), schizophrenia (*P* = 4.7 x 10^-17^), and bipolar disorder (*P* = 9 x 10^-6^) had a younger age than expected, and young genes showed significantly higher expression in the pars triangularis (*P* = 1.4 x 10^-4^).

***LoF genes.*** The top 10% of genes (*n* = 1,253 genes) with the highest and lowest Loss-of-Function (LoF) scores (Balasubramanian et al. 2017) were extracted from 12,526 genes with available LoF and dating estimates. An independent two-sided t-test compared the median evolutionary age estimates between these two groups (Figure 4A). The same test was conducted with varying gene thresholds (top 20%, 5% and 1%; see *Validation LoF gene analysis* in Supplementary Results).

***Gene-analysis.*** Gene-analysis to identify the genes involved with brain, cognition and neuropsychiatric phenotypes was conducted using MAGMA (v1.09) (De Leeuw et al. 2015) with Phase 3 of the 1,000 Genomes reference panel (European population, GRCh37) on raw GWAS summary statistics for schizophrenia (Ruderfer et al. 2018), bipolar disorder (Ruderfer et al. 2018), major depressive disorder (eQTLGen et al. 2018), autism spectrum disorder (Autism Spectrum Disorder Working Group of the Psychiatric Genomics Consortium et al. 2019), and Alzheimer’s disease (Jansen et al. 2019). Additionally, five major brain and cognitive phenotypes, including brain volume (Jansen et al. 2020), cortical area (Grasby et al. 2020), cortical thickness (Grasby et al. 2020), intelligence (Savage et al. 2018), and social behavior (Day et al. 2018) (see Table S9 for details on included studies). FDR-significant genes (*q* < 0.05) were included for further analysis.

***Functional gene annotation.*** Functional annotation of the young genes was conducted using FUMA (v1.5.2) (Watanabe et al. 2017), a web-based platform for functional gene annotation integrating multiple biological resources (https://fuma.ctglab.nl/). FUMA conducts gene-based pathway enrichment analyses by comparing the prioritised genes against GO biological function gene sets, using hypergeometric tests to assess the overrepresentation (*q* < 0.05, FDR). Enrichment analysis of the youngest 10% of genes was conducted using protein-coding gene sets as the background for the hypergeometric test and excluding the MHC region.

***Cortical gene expression.*** Cortical gene microarray transcriptome data were obtained from the Allen Human Brain Atlas (AHBA; http://human.brain-map.org/static/download), which includes gene expression profiles from brain samples of six human donors (five males, one female) without a history of neuropsychiatric or neuropathological conditions. The dataset consists of expression levels for 20,734 genes measured by 58,692 probes across cortical regions of the left hemisphere (Hawrylycz et al. 2012). Tissue samples were mapped to cortical areas using the FreeSurfer Desikan Killiany atlas (Desikan et al. 2006) (DK, *n* = 34 left cortical areas) based on their proximity to the nearest voxel within the cortical ribbon of MNI 152 template. Gene expression data were z-normalized, averaged across regions, and then across subjects, resulting in a group-level gene expression matrix (34 x 20,734 genes), for more details in the preprocessing pipeline see (Wei et al. 2022). For validation, AHBA left-hemisphere samples were also mapped using von Economo-Koskinas cortical type atlas (Pijnenburg et al. 2021) (EK, *n* = 15 regions), Brodmann Atlas (Pijnenburg et al. 2021) (BA, *n* = 39 regions), and 114-region DK subdivision (Cammoun et al. 2012) (DK-114, *n* = 57 regions).

RNA sequencing data from the BrainSpan Atlas of the Developing Human Brain (Miller et al. 2014) were used to investigate the gene expression across the lifespan. This dataset contains 52,376 gene-level RPKM values from 391 cortical samples across 42 postmortem human brains, covering 31 developmental stages ranging from 8 weeks post-conception to 40 years old. Cortical samples were averaged across subjects to generate a group-level expression matrix (time periods x genes). Genes with both expression and evolutionary age data (*n* = 13,502) were included in further analyses. The median expression of the youngest 10% of genes (*n* = 1,351, evolutionary age: 54,080 to 3,803 years) and the oldest 10% (n = 1,351, evolutionary age: 2,965,600 to 353,462 years) was computed for each developmental stage. A two-sided t-test was used to assess whether prenatal expression levels (8 to 37 weeks post-conception) were significantly higher or lower than postnatal expression levels (4 months to 40 years old).

***Gene expression association with cognitive domains.*** Gene expression patterns of young genes were analyzed for overexpression in regions associated with cognitive functions using the Neurosynth database (www.neurosynth.org), a comprehensive meta-analysis of human brain functional data from over 14,000 MRI studies (Yarkoni et al. 2011). Voxel-wise *z*-scores, indicating the association of each voxel with a cognitive term (*n* = 111 terms; FDR corrected, *q* < 0.01), were mapped to the MNI152 template in FreeSurfer space. The median *z*-score was calculated for each left hemisphere cortical region within the 114-region DK subdivision, and terms with regions exhibiting a *z*-score > 2 were included in the analysis. The median expression of young genes was then estimated within these regions and tested for enrichment through null-random-gene permutation testing (see *Gene expression analysis* in Materials and Methods) (Wei et al. 2022). A follow-up analysis investigated gene expression patterns in ‘language network’ regions across evolutionary stages by ordering genes based on age, dividing them into ten bins (1,635 genes each), and assessing differential cortical expression across these bins.

**Supplementary Results**

**SNP-level analyses**

***MAF evolutionary age analysis.*** We examined the genetic timeline of high and low MAF variants due to their dominant role in human traits and disorders (Watanabe et al. 2019). Phenotype-associated SNPs were categorized into high MAF (MAF ≥ 0.4, *n* = 7,581 SNPs) and low MAF (MAF ≤ 0.1, *n* = 6,281 SNPs) groups, and the proportion of each group appearing within old and young peaks was evaluated. High MAF variants were predominantly found in the old peak (83.8% of all high MAF variants, *P* = 4.6 x 10^-193^), while low MAF variants were overrepresented in the young peak (60.1% of all low MAF variants, *P* < 1 x 10^-324^; Figure 1B).

***Phenotype-associated SNPs timeline replication.*** To validate the distinctiveness of the bimodal distribution of phenotype-associated SNPs extracted from the GWAS Atlas (Watanabe et al. 2019), its genetic timeline was compared to the distribution of SNP evolutionary ages from the EBI Catalog (Buniello et al. 2019), which includes 88,362 unique SNPs that do not overlap with the GWAS Atlas. The time dimension of both datasets was divided into 100 bins, and the number of SNPs within each bin was counted. Spearman’s correlation was used to assess the similarity between the SNP count distributions of both datasets. The bimodal distribution observed in the GWAS Atlas closely matched that of the EBI Catalog (rho = 0.98, *P* = 1.5 x 10^-72^; Figure 1C).

***Phenotype evolutionary age***

*Chapter level.* We analyzed phenotype-associated SNPs linked to modern human traits at a higher phenotypic resolution. Specifically, we assessed whether the evolutionary age of phenotypes at the chapter level was significantly older or younger than expected, accounting for polygenicity and MAF (for the domain level, see *Cognitive and psychiatric phenotypes are shaped by recent genetic modifications* in Results). At the chapter level (*n* = 31 chapters, Bonferroni *P* < 1.6 x 10^-3^; Figure 2C), SNPs related to 'Mental and Behavioral Disorders' (*n* = 1,893 SNPs, median evolutionary age = 83,250 years old, *P* = 1.6 x 10^-68^) and 'Major Life Areas' (*n* = 817, median age = 617,780, *P* = 6.7 x 10^-5^) displayed an evolutionary age younger than expected. The oldest chapters were 'Malignant Neoplasms' (*n* = 363, median age = 590,657, *P* = 6.7 x 10^-6^), 'Functions of the Digestive, Metabolic and Endocrine Systems' (*n* = 10,147, median age = 785,400, *P* = 1.5 x 10^-4^; see Table S2 for complete chapter results).

*Subchapter level.* At the subchapter level (*n* = 75 subchapters, Bonferroni *P* < 6.7 x 10^-4^; Figure S1), the phenotypes that displayed an age younger than expected were 'Mental and Behavioural Disorders Due to Use of Alcohol' (*n* = 481, median age = 39,548, P = 5.2 x 10^-12^), 'Sexual functions' (n = 349, median age = 76,814, P = 7.7 x 10^-12^), 'Looking After One's Health' (n = 1,359, median age = 646,077, P = 2.3 x 10^-5^), 'Education' (n = 578, median age = 637,646, P = 2.5 x 10^-5^), and 'Depressive Episode' (*n* = 658 SNPs, median evolutionary age = 24,470 years old, *P* = 1.6 x 10^-4^). Subchapters 'Malignant Neoplasms of Breast' (*n* = 215, median age = 487,202, *P* = 1.4 x 10^-16^), 'General Metabolic Functions' (n = 1043, median age = 667,462, *P* = 5.3 x 10^-6^) were found to show an age significantly older (see Table S2 for complete subchapters results).

*Trait level.* Zooming into the individual trait level (*n* = 361 traits, Bonferroni *P* < 1.4 x 10^-4^; Figure 2A, S2) we found that 'Use of sun/uv protection' was significantly younger after Bonferroni correction (*n* = 28 SNPs, median evolutionary age = 185,084 years old, *P* = 1.7 x 10^-5^), followed by 'Educational attainment' (*n* = 465, median age = 637,522, *P* = 1.3 x 10^-4^), and 'Fluid intelligence score' (*n* = 58, median age = 505,506, *P* = 1.4 x 10^-4^). We also observed a trend toward a younger evolutionary age (nominally significant) in phenotypes related to psychiatric disorders such as 'Schizophrenia/Bipolar disorder' (*n* = 86, median age = 499,765, *P* = 2.8 x 10^-4^), 'Depression - Lifetime number of depressed periods' (*n* = 352, median age = 19,938, *P* = 1.3 x 10^-3^), 'Average weekly intake of other alcoholic drinks' (*n* = 252, median age = 19,506, *P* = 1.1 x 10^-2^), 'Depression' (*n* = 40, median age = 536,490, *P* = 1.1 x 10^-2^). The top traits with the (nominally significant) oldest age were 'Ulcerative colitis' (*n* = 226, median age = 594,015, P = 1.5 x 10^-3^), 'Ease of skin tanning' (*n* = 319, median age = 628,330, P = 2.3 x 10^-3^), 'Getting up in morning' (*n* = 52, median age = 1,038,246, *P* = 9.6 x 10^-3^), 'Waist circumference (adjusted for BMI)' (*n* = 89, median age = 1,036,332, *P* = 2.1 x 10^-1^; see Table S2 for complete traits results).

*Validation phenotype evolutionary age.* To ensure the robustness of our results across different studies and SNP sets, we repeated the main analysis at the domain and trait level, applying three simultaneous filtering procedures: 1) including only linkage disequilibrium (LD) independent SNPs (R^2^ < 0.1) and excluding those within the major histocompatibility complex (MHC); 2) including GWAS with a sample size above 50,000 subjects; 3) including SNPs with a date quality score above 0.7, a reliability metric (ranging from 0-1) provided by the HGD database.

At the domain level (*n* = 13 domains, Bonferroni *P* value threshold < 3.8 x 10^-3^), only 'Psychiatric' had a significantly younger age (*n* = 498 SNPs, 21,278 years; *P* = 6.6 x 10^-7^). Domains with significantly older age were 'Dermatological' (*n* = 133, 57,399 years; *P* = 7.9 x 10^-25^), and 'Endocrine' (*n* = 38, 387,934 years; *P* = 1.7 x 10^-5^). At the trait level (*n* = 37 traits, Bonferroni *P* < 1.4 x 10^-3^), significantly younger phenotypes included 'Depression - Lifetime number of depressed periods' (*n* = 203, 17,624 years; *P* = 8 x 10^-8^), followed by 'Average weekly intake of other alcoholic drinks' (*n* = 156, 17,944 years; *P* = 2.3 x 10^-5^), and 'Lifetime number of sexual partners' (*n* = 99, 17,445 years; *P* = 2.9 x 10^-4^). The only trait with a significantly older evolutionary age was 'Ease of skin tanning' (*n* = 53, 57,003 years, *P* = 1.7 x 10^-41^).

To further account for LD effects, we applied the same three filters as before while including only SNPs with no LD dependencies (regardless of the R^2^) within the same phenotype. At the domain level (n = 20 domains, Bonferroni *P* < 2.5 x 10^-3^), only SNPs related to 'Psychiatric' (*P* = 2.1 x 10^-8^) had a younger age than average, while ‘Dermatological’ SNPs were older than average (*P* = 5.4 x 10^-7^). At the trait level (*n* = 198 traits, Bonferroni *P* < 2.5 x 10^-4^) the youngest phenotypes were 'Depression - Lifetime number of depressed periods' (*P* = 8.7 x 10^-8^), 'Average weekly intake of other alcoholic drinks' (*P* = 7.0 x 10^-6^), and 'Lifetime number of sexual partners' (*P* = 9.8 x 10^-5^). We also observed nominally significant effects toward younger ages in other cognitive and psychiatric phenotypes, including 'Ever smoker' (*P* = 0.013), 'Intelligence' (*P* = 0.014), 'Well-being spectrum' (*P* = 0.020), 'Educational attainment' (*P* = 0.022), and 'Schizophrenia' (*P* = 0.026). Only the trait 'Ease of skin tanning' (*P* = 5.8 x 10^-8^) showed a significantly older age than average.

*Replication phenotype evolutionary age using the EBI Catalog*. We aimed to replicate the main analysis by estimating the expected evolutionary age of human phenotypes while controlling for polygenicity and MAF, using the EBI Catalog (Buniello et al. 2019). We analysed GWAS not included in the GWAS Atlas (*n* = 465 phenotypes, Bonferroni *P* < 1.1 x 10^-4^). The phenotypes showing an evolutionary age significantly younger were 'Post bronchodilator FEV1' (*n* = 506 SNPs, median evolutionary age = 268,138 years old, *P* = 1.3 x 10^-30^), 'Itch intensity from mosquito bite adjusted by bite size' (*n* = 123, 68,137 years, *P* = 4 x 10^-8^), 'Deliberate self-harm' (*n* = 73, 287,165 years, *P* = 3.2 x 10^-5^), 'Interleukin-18 levels' (*n* = 34, 110,000 years, *P* = 4.2 x 10^-5^), 'General cognitive ability' (*n* = 870, 643,425 years, *P* = 5.9 x 10^-5^), 'Total body bone mineral density' (*n* = 122, 461,802 years, *P* = 7.1 x 10^-5^), and 'Cognitive aspects of educational attainment' (*n* = 103, 483,570 years, *P* = 9.1 x 10^-5^). The phenotypes with an older age than expected were 'Adolescent idiopathic scoliosis' (*n* = 842, 742,063 years, *P* = 3.2 x 10^-12^), 'Suicidal ideation' (*n* = 26, 1,254,047 years, *P* = 2.4 x 10^-9^), 'Cerebrospinal fluid t-tau:AB1-42 ratio' (*n* = 25, 852,640 years, *P* = 2.5 x 10^-5^), 'Trans fatty acid levels' (*n* = 91, 864,825 years, *P* = 6 x 10^-5^), and 'IgG glycosylation' (*n* = 349, 851,647 years, *P* = 7.5 x 10^-5^; see Table S3 for complete results).

**Genetic timeline of brain-imaging phenotypes**

***BRAIN-SNPs timeline.*** The genetic timeline of BRAIN-SNPs (see *BRAIN-SNPs* in Supplementary Methods) revealed four time periods with a significantly higher number of SNPs (*n* = 100 bins of ~20,000 years, Bonferroni *P* < 5 x 10^-4^; see *Temporal analysis* in Material and Methods). Two time bins showed significantly increase SNPs at the beginning of the old peak: 1,980,075 to 1,960,150 years ago (*P* = 2.9 x 10^-6^), and 1,880,475 to 1,860,550 years ago (*P* = 7.4 x 10^-5^). Two time bins coincided with the highest point of the young peak: 47,625 to 27,700 years ago (*P* = 1.4 x 10^-8^), and 27,700 to 7,775 years ago (*P* = 2.1 x 10^-21^; Figure S4).

***BRAIN-SNPs evolutionary age*.** Statistical analysis (controlling for the number of variants linked to brain structure and MAF distribution; see *Evolutionary age of human phenotypes* Materials and Methods) revealed that genetic variants related to the cortex (*n* = 126 SNPs, 400,170 years) showed a borderline (non-significant) effect towards younger age than average (*P* = 0.06; Figure S5A). In contrast, variants associated with the caudate nucleus (*n* = 22 SNPs, 1,095,415 years) exhibited a nominally older evolutionary age (*P* = 0.029), though this effect did not survive multiple comparison correction. No specific effects were observed for variants linked to white matter (*n* = 627 SNPs), amygdala (*n* = 37 SNPs), hippocampus (*n* = 29 SNPs), or thalamus (*n* = 17 SNPs), among others brain structures (see Table S6 for complete results).

To further validate these findings, alternative GWAS summary statistics were analyzed for seven brain structures (Bonferroni *P* < 7.1 x 10^-3^). Genome-wide significant SNPs (*P* < 5 x 10^-8^) associated with cortical surface (*n* = 827 SNPs, 289,780 years) and thickness (*n* = 296 SNPs, 279,068 years) were significantly younger age than average when controlling for polygenicity and MAF distribution (*P* = 9.1 x 10^-72^ and 4 x 10^-28^, respectively). In contrast, hippocampus (*n* = 26 SNPs, 508,314 years, *P* = 1.5 x 10^-15^) and cerebellum SNPs (*n* = 1,281, 764,970 years, *P* = 7.4 x 10^-10^) exhibited significantly older ages.

***BRAIN-SNPs located within human accelerated regions*.** BRAIN-SNPs were analyzed for their presence in genes located within human accelerated regions (HAR)—genomic regions uniquely conserved in other species but divergent in humans (see *Human accelerated regions* in Supplementary Methods) (Pollard et al. 2006). Cortex-related genetic variants showed the highest overlap with HAR genes (19% of cortex-related SNPs), significantly exceeding expectations based on SNP count, evolutionary age, and MAF (*P* < 1 x 10^-10^). Other brain structures also exhibited significant HAR gene overlap: cerebellum (17% of SNPs, *P* < 1 x 10^-10^), amygdala (16% of SNPs, *P* = 3.7 x 10^-3^), and white matter (13% of SNPs, *P* < 1 x 10^-10^), while thalamus-related variants had the lowest overlap with HAR genes (0.06% of SNPs; *P* > 0.05).

***BRAIN-SNPs timeline co-fluctuation.*** We investigated whether the BRAIN-SNPs associated with the cortical followed a similar or distinct genetic timeline compared to SNPs linked to other brain structures (Barton and Harvey 2000; Whiting and Barton 2003). The genetic timeline of cortical SNPs (*n* = 100 bins; 36 tests, Bonferroni *P* < 1.4 x 10^-3^; excluded SNPs overlapping between structures; see *BRAIN-SNPs timeline co-fluctuation* in Supplementary Methods) showed the strongest correlation with the genetic timeline of white matter SNPs (Spearman's *rho* = 0.72, *P* = 3.9 x 10^-17^; Figure S5B). Cortical SNPs timeline also exhibited significant overlap with cerebellum (*rho* = 0.70, *P* = 7.1 x 10^-16^) and hippocampus (*rho* = 0.52, *P* = 3.7 x 10^-8^). In contrast, cortical SNPs showed the lowest correlation with the genetic timeline of thalamus-related SNPs (*rho* = 0.28, *P* = 4 x 10^-3^), supporting evidence of distinct evolutionary patterns between cortical and subcortical areas (Barton and Harvey 2000; Tuller et al. 2008). SNPs linked to cerebellum variation exhibited significant co-fluctuation with all other brain structures, with correlations ranging from 0.37 (thalamus, *P* = 1.4 x 10^-4^) to 0.80 (white matter, *P* = 5.3 x 10^-24^; see Table S7 for all correlations).

***Validation BRAIN-SNPs timeline co-fluctuation.*** We assessed the co-fluctuation of SNP occurrence across time for each brain structure (bins = 100; 36 tests, Bonferroni *P* < 1.4 x 10^-3^). To validate the main results, the analysis was repeated including only SNPs younger than 2 million years. SNPs related to the cortex showed the highest correlations with cerebellum (*rho* = 0.36, *P* = 2.7 x 10^-4^) and white matter (*rho* = 0.32, *P* = 1.2 x 10^-3^), and the lowest non-significant correlations with pallidum (*rho* = 0.04, *P* > 0.05), nucleus accumbens (*rho* = 0.16, *P* > 0.05), and thalamus (*rho* = 0.19, *P* > 0.05). The genetic timeline of cerebellum SNPs was significantly correlated with white matter (*rho* = 0.54, *P* = 8.8 x 10^-9^) and hippocampus (*rho* = 0.36, *P* = 1.9 x 10^-4^); amygdala correlated with white matter (*rho* = 0.33, *P* = 9.1 x 10^-4^); and nucleus accumbens correlated with pallidum (*rho* = 0.37, *P* = 1.8 x 10^-4^).

**Gene-level analysis**

***Validation LoF gene analysis****.* An independent t-test was conducted to compare the median evolutionary age between the most intolerant and tolerant Loss-of-Function (LoF) genes (Balasubramanian et al. 2017) across varying gene set sizes. The top 20% intolerant LoF genes were significantly younger than the top 20% tolerant LoF genes (*n* = 2,505; *t* = -4.75, *P* = 2 x 10^-6^). Similar results were found for the top 5% (*n* = 627; *t* = -3.16, *P* = 1.5 x 10^-3^), and top 1% LoF genes (*n* = 125; *t* = -2.72, *P* = 6.9 x 10^-3^; Bonferroni *P* < 1.7 x 10^-2^).

To account for potential biases, gene length was regressed out of the median evolutionary age, and the main analysis was repeated. The findings remained robust, confirming that the top 10% intolerant LoF genes were significantly younger than tolerant LoF genes (*t* = -4.78, *P* = 1.9 x 10^-6^).

***Validation gene-set analysis.*** We repeated the gene-set analysis (see *Gene-set analysis* in Materials and Methods and Table S9 for included GWAS) using MAGMA (De Leeuw et al. 2015), varying the size of the oldest and youngest gene sets to test for enrichment. The youngest 20% genes (*n* = 3,666 genes) were enriched for intelligence (*b* = 0.15, *P* = 5.5 x 10^-14^), schizophrenia (*b* = 0.10, *P* = 3 x 10^-6^), cortical thickness (*b* = 0.05, *P* = 1.8 x 10^-3^). No enrichment was found for the oldest 20% genes for any phenotype (*P* > 0.05). The youngest 5% of genes (*n* = 917 genes) showed enrichment for intelligence (*b* = 0.11, *P* = 4.7 x 10^-3^), cortical area (*b* = 0.08, *P* = 1.5 x 10^-2^) and schizophrenia (*b* = 0.09, *P* = 1.3 x 10^-2^), although these effects did not surpass Bonferroni correction. No enrichment was found for the oldest 5% genes for any phenotype (*P* > 0.05). No significant enrichment was observed when including the top 1% youngest and oldest genes (*n* = 183 genes; *P* > 0.05).

The top 10% youngest genes (adjusted for median evolutionary age while controlling for gene length) showed enrichment for intelligence (*b* = 0.11, *P* = 7.3 x 10^-5^), and cortical area (*b* = 0.08, *P* = 1.2 x 10^-3^). No enrichment was found for the oldest 10% genes for any phenotype (*P* > 0.05).

***Validation phenotype evolutionary genes age.*** To account for differences in the number of genes associated with each phenotype across GWAS (identified via gene analysis), we further validated the main results by selecting the 200 genes with the largest z-statistic from the MAGMA gene analysis (De Leeuw et al. 2015) for each GWAS (ten tests, Bonferroni *P* < 5 x 10^-3^). The top genes associated with brain, cognitive and neuropsychiatric phenotypes that had significantly younger age were intelligence (*P* = 2.0 x 10^-4^), brain volume (*P* = 1.7 x 10^-4^), sociability (*P* = 2.4 x 10^-4^), schizophrenia (*P* = 1.2 x 10^-3^) and Alzheimer’s disease (*P* = 2 x 10^-3^).

We further validated the main analysis by selecting genes with more than 10 SNPs and a median date quality score above 0.7 (*n* = 14,523 genes). Again, genes with a significantly younger evolutionary age included intelligence (*P* = 1.1 x 10^-17^), schizophrenia (*P* = 1.6 x 10^-15^), bipolar disorder (*P* = 6.5 x 10^-5^), brain volume (*P* = 9.1 x 10^-5^), sociability (*P* = 1.5 x 10^-4^), and Alzheimer’s disease (*P* = 2.5 x 10^-3^).

To ensure gene length did not bias the results, we regressed out gene length from evolutionary age and repeated the analysis. The findings remained robust, showing that genes related to intelligence (*P* = 1.5 x 10^-13^), schizophrenia (*P* = 4.2 x 10^-11^), brain volume (*P* = 8.1 x 10^-5^), bipolar disorder (*P* = 1.6 x 10^-4^), and Alzheimer’s disease (*P* = 3.4 x 10^-3^) were significantly younger than average.

***Validation gene expression permutation test (1).*** We examined whether the top 10% youngest genes are significantly highly or lowly expressed in specific brain regions using different cortical atlases. The EK atlas (Pijnenburg et al. 2021) provided information about brain cytoarchitectonics (15 brain regions, Bonferroni *P* < 3.3 x 10^-3^), revealing a significantly higher expression in the agranular medial orbitofrontal (*P* = 2 x 10^-6^), and a lower expression in the polar occipital cortex (*P* = 8 x 10^-4^). Using BA atlas (Pijnenburg et al. 2021) (39 brain regions, Bonferroni *P* < 1.2 x 10^-3^), young genes showed a higher expression in BA45 (*P* = 3 x 10^-4^) and lower expression in BA5 (*P* = 3 x 10^-4^). Using DK-144 atlas (Cammoun et al. 2012) (57 brain regions, Bonferroni *P* < 8.7 x 10^-4^), we found a nominally higher expression in rostral middle frontal (*P* = 1 x 10^-3^), superior parietal (*P* = 4.6 x 10^-3^), postcentral (*P* = 5.9 x 10^-3^) and pars triangularis (*P* = 7.8 x 10^-3^), and lower expression in lateral occipital cortex (*P* = 1.4 x 10^-3^), though these effects did not survived Bonferroni correction.

***Validation gene expression permutation test (2).*** To assess the robustness of the results, we repeated the main analysis while varying the number of young genes included in the gene expression analysis. The top 20% youngest genes (*n* = 3,269 genes, 69,633 to 6,717 years ago) were nominally overexpressed in the caudal anterior cingulate (*P* = 0.023), but did not surpass Bonferroni correction. The 5% youngest genes (*n* = 818 genes, 44,157 to 6,717 years ago) were nominally overexpression in language-related areas, namely pars opercularis (*P* = 3.5 x 10^-3^), banks of the superior temporal sulcus (*P* = 7.6 x 10^-3^), and pars triangularis (*P* = 1.3 x 10^-2^), however these effects were no significant after Bonferroni correction. The 1% youngest genes (*n* = 164, 26,552 to 6,717 years ago) showed a significant overexpression in the pars triangularis (*P* = 6.2 x 10^-4^).

***Validation gene expression permutation test (3).*** We repeated the main analysis using the DK atlas (34 brain regions, Bonferroni *P* < 1.4 x 10^-3^), restricting the analysis to genes with more than 10 SNPs and a median quality score above 0.7 (*n* = 13,852 genes with gene expression and evolutionary age information). The top 10% youngest genes (*n* = 1,386) were nominally overexpressed in posterior cingulate (*P* = 4.5 x 10^-2^). The top 5% youngest genes (*n* = 693) showed no differential expression across regions (*P* > 0.05). The top 1% youngest genes (*n* = 139) were nominally overexpressed in pars triangularis (P = 7.1 x 10^-3^), but significance level did not surpass Bonferroni threshold.

After regressing out gene length from median evolutionary age, the top 10% youngest genes showed a significantly higher expression in pars triangularis (*P* = 9.8 x 10^-5^).

**Supplementary Figures**


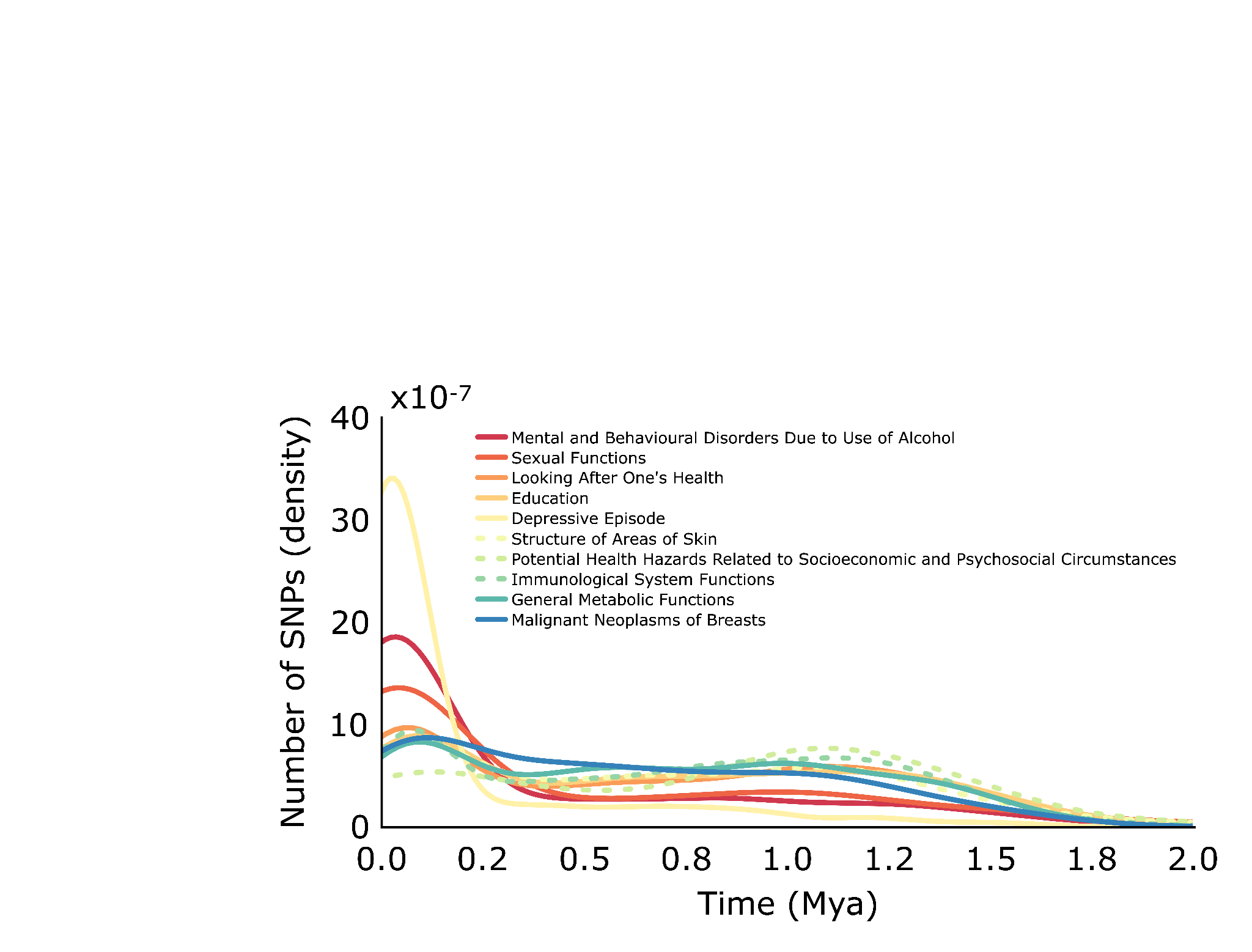


**Supplementary Figure 1.** **Genetic timeline of phenotype-associated SNPs (subchapter level).**

Timeline of SNPs related to human phenotypes at the subchapter level from the GWAS Atlas. Histogram of the density (y-axis) of the number of SNPs emerging across time (shown until 2M years ago; x-axis). Figure shows the top five subchapters with a median evolutionary age younger (following legend order, from 'Mental and Behavioural Disorders Due to Use of Alcohol' to 'Depressive Episode') and older (from 'Structure of Areas of Skin' to 'Malignant Neoplasms of Breasts') than expected by the null model (controlling for polygenicity and MAF; *n* = 75 subchapters, Bonferroni *P* value threshold < 6.7 x 10^-4^). Dotted lines denote nominal effects, while solid lines indicate Bonferroni significant effects. Mya, million years ago.


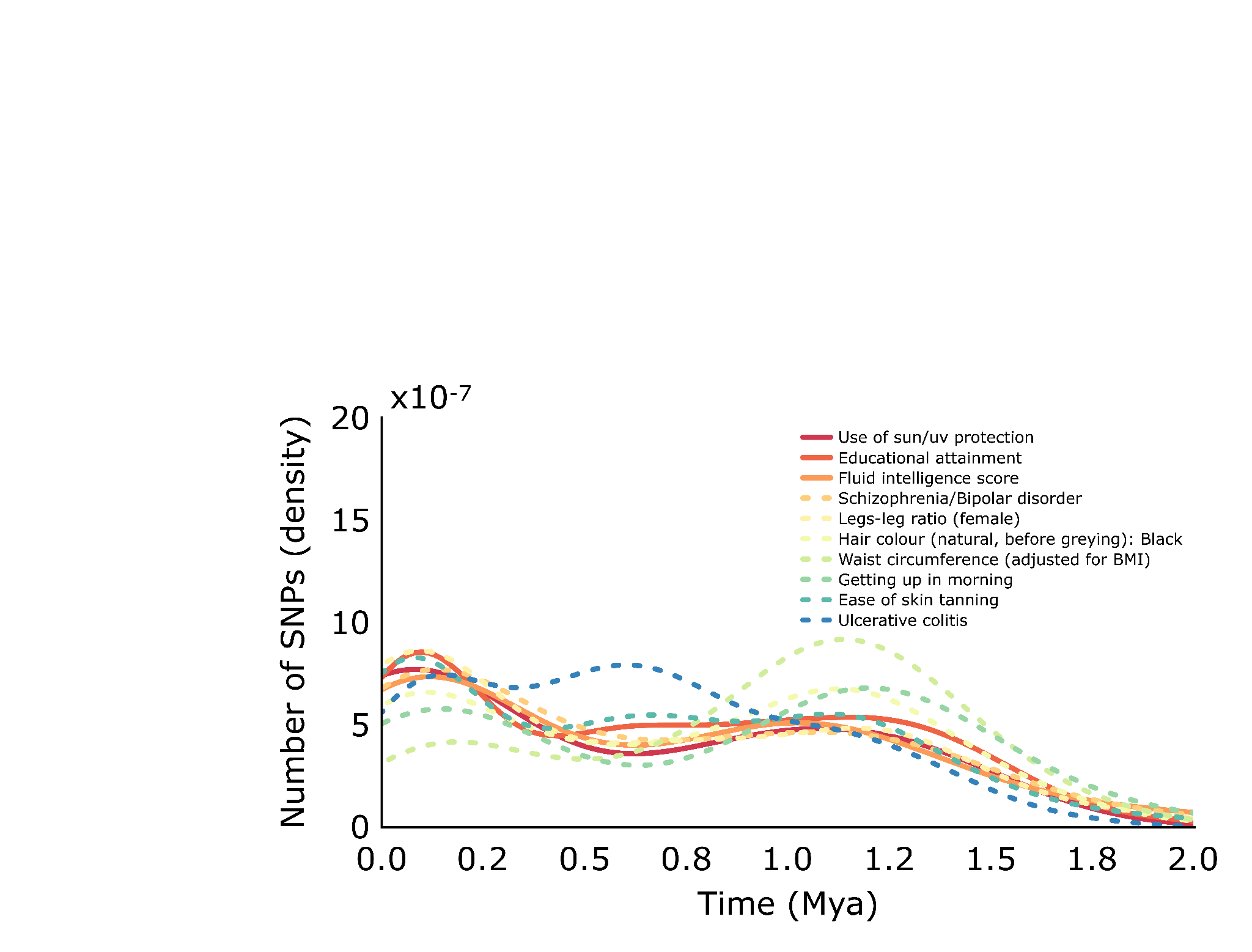


**Supplementary Figure 2.** **Genetic timeline of phenotype-associated SNPs (trait level).**

Timeline of SNPs related to human phenotypes at the trait level from the GWAS Atlas. Histogram of the density (y-axis) of the number of SNPs emerging across time (shown until 2M years ago; x-axis). Figure shows the top five traits with a median evolutionary age younger (following legend order, from 'Use of sun/uv protection' to 'Legs-leg ratio (female)') and older (from 'Hair colour (natural, before greying): Black' to 'Ulcerative colitis') than expected by the null model (controlling for polygenicity and MAF; *n* = 361 traits, Bonferroni *P* value threshold < 1.4 x 10^-4^). Mya, million years ago.


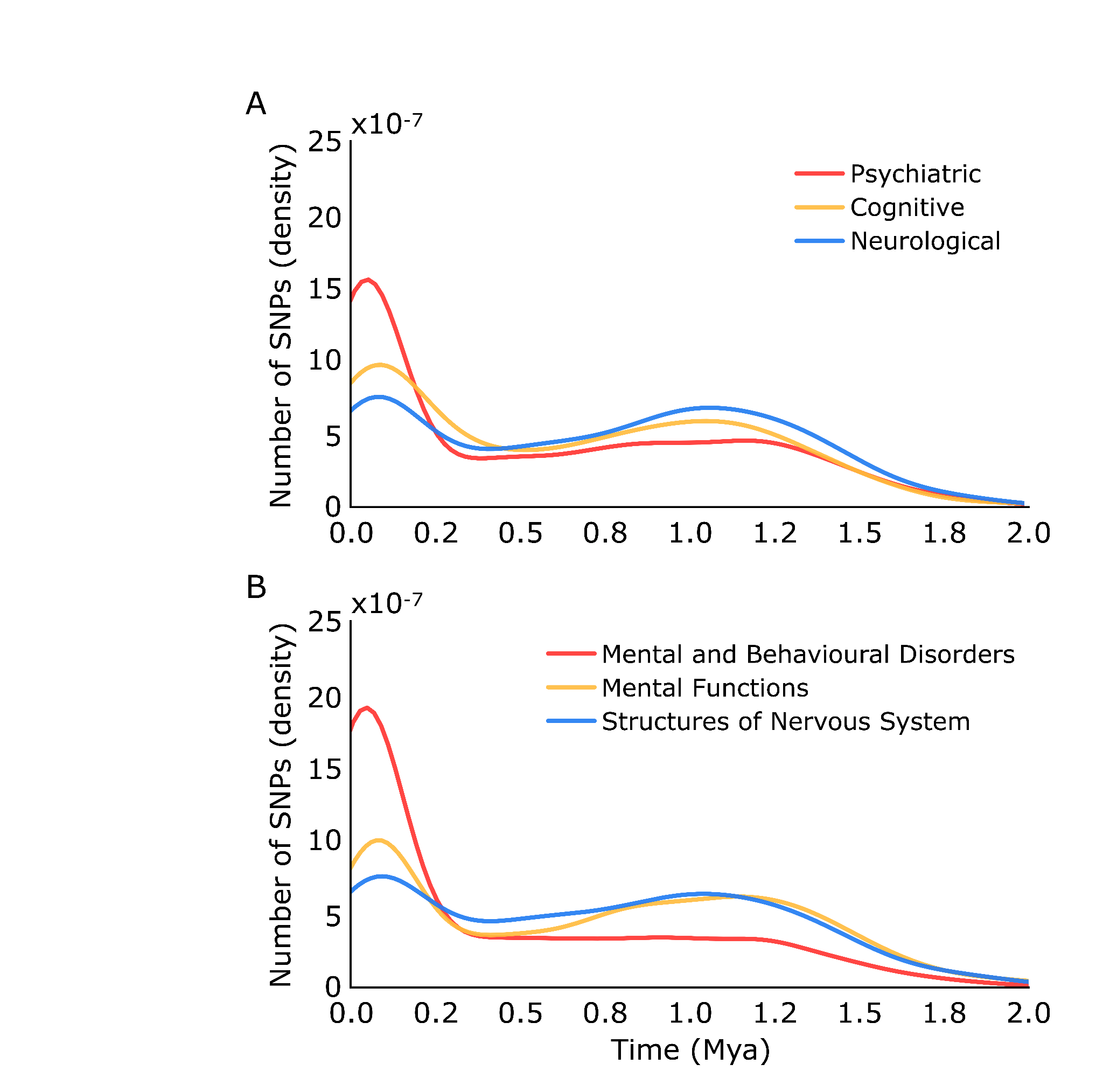


**s**

**Supplementary Figure 3. Genetic timeline of brain, cognitive and psychiatric traits.** Timeline of the density (normalised count; y-axis) of the number of SNPs across the last 2M years (x-axis) in relation to brain-related traits (blue), cognition (yellow) and psychiatric disorders (red). The phenotypes are organised at the (*A*) domain and (*B*) more detailed chapter levels. Genetic variants related to the neurological domain (median evolutionary age = 800,850 years old) emerged earlier in evolution than variants related to cognition (*t* = 2.70, *P* = 7 x 10^-3^; median age = 682,673) and psychiatric disorders (*t* = 7.29, *P* = 3.7 x 10^-13^; median age = 475,832). Mya, million years ago.


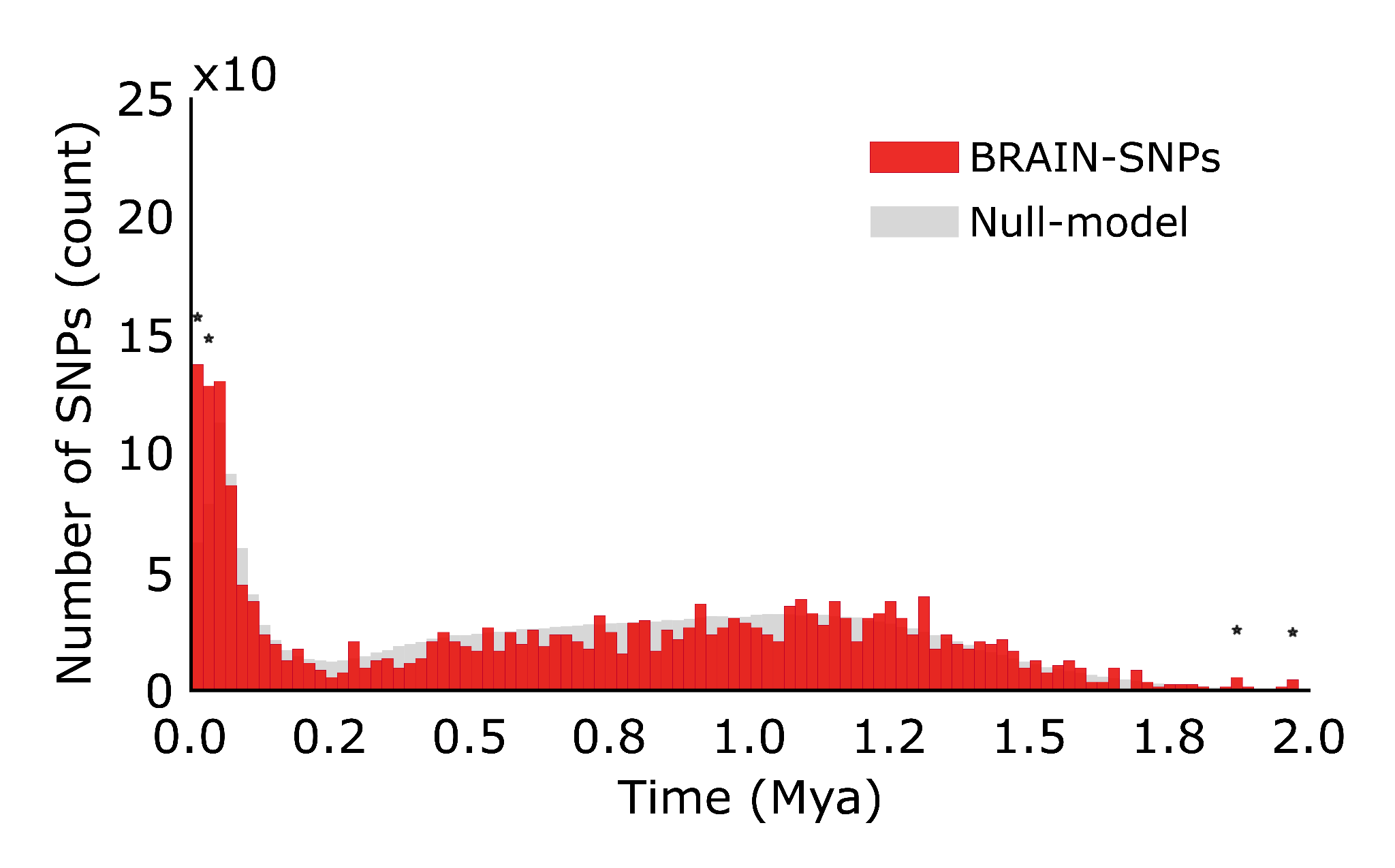


**Supplementary Figure 4. Genetic timeline of BRAIN-SNPs.**

Absolute count (y-axis) of number of SNPs per time-bin (100 bins of ~20,000 years old; shown until 2M years ago; x-axis) of all significant BRAIN-SNPs (red) associated with brain phenotypes extracted from the UK Biobank BIG40 (BRAIN-SNPs, 2,273 unique SNPs). BRAIN-SNPs date estimates ranged from 3,621,625 to 4,904 years ago. Asterisks (*) denote bins where the number of BRAIN-SNPs significantly exceed the null model of random equally sized sets of SNPs selected from all SNPs from the HGD (100 tests, Bonferroni *P* value threshold < 5 x 10^-4^). Mya, million years ago.


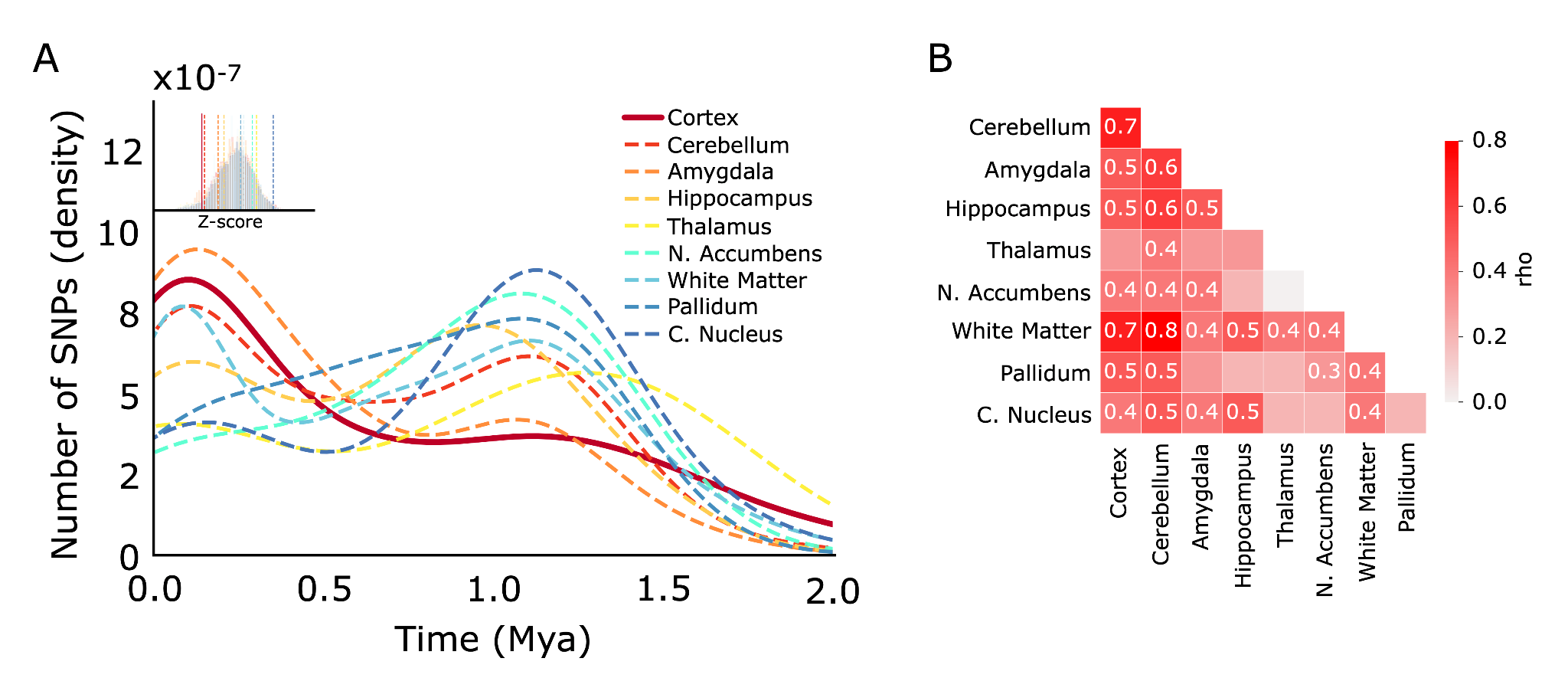


**Supplementary Figure 5. Genetic timeline of brain-imaging phenotypes.** (A) Timeline of brain structures. Plots show the density distribution (y-axis) of the number of SNPs per age bin (x-axis). Solid red line highlights cortex SNPs (see insert for the null model of each brain phenotype). (B) Heatmap of the co-fluctuation between the timelines of SNPs related to brain structures (Spearman's correlation). Only significant correlations are shown (Bonferroni *P* < 1.4 x 10^-3^). Mya, million years ago; C. Nucleus, caudate nucleus; N. Accumbens, nucleus accumbens.


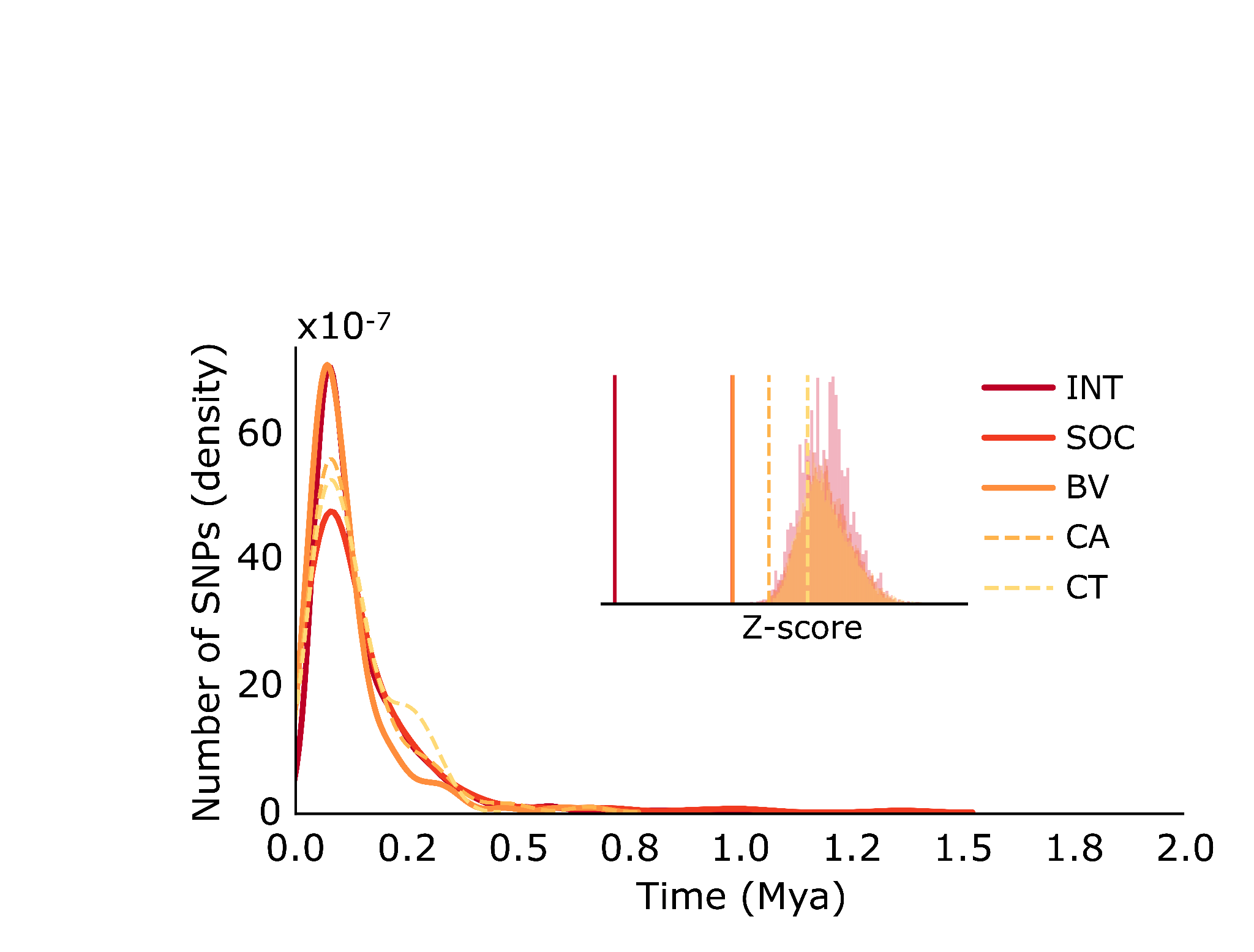


**Supplementary Figure 6. Genetic timeline of genes linked to brain and cognitive phenotypes.**

Timeline of genes associated with five brain and cognitive phenotypes, including intelligence, sociability, brain volume, cortical area, and cortical thickness from previous GWAS. Histogram of the density (y-axis) of the number of genes emerging across time (shown until 2M years ago; x-axis). Solid lines denote phenotypes significantly younger than the null model (Bonferroni *P* < 5 x 10^-3^; see insert for the null model distribution of each condition). Mya, million years ago; INT, intelligence; SOC, sociability; BV, brain volume; CA, cortical area; CT, cortical thickness.


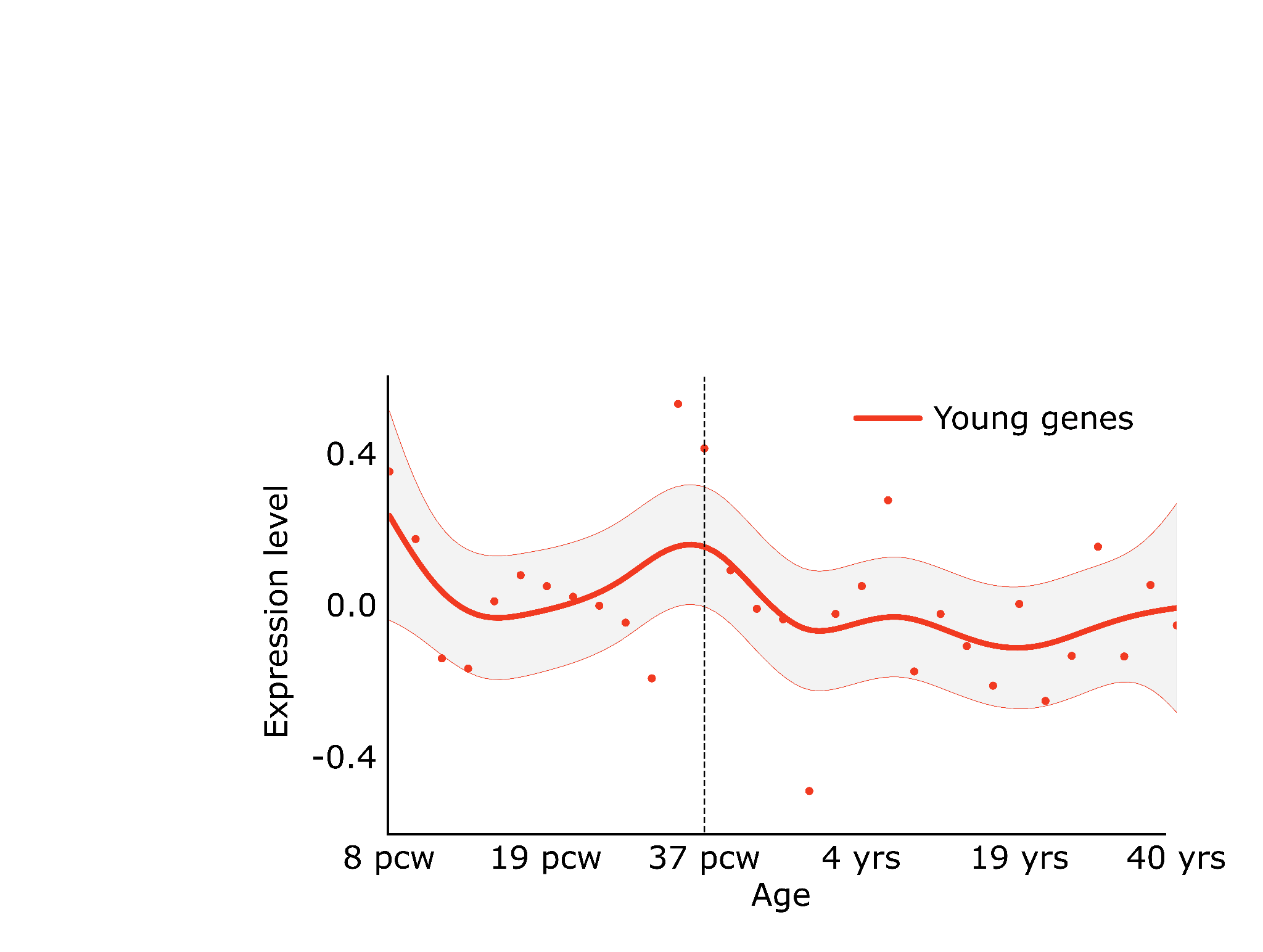


**Supplementary Figure 7. Transcriptomic developmental trajectory of evolutionarily recent genes.** The expression levels trajectory of the top 10% youngest genes across the lifespan from 8 weeks post-conception to 40 years old (x-axis). For visualisation purposes, we normalised the expression of each gene across time, shown as *z*-scores (y-axis). Dots indicate the normalised mean expression of the youngest genes at each developmental stage, while the line and shadow represent the predicted value using a generalised additive model and the corresponding uncertainty, respectively. Dotted line distinguishes prenatal from postnatal stages. *pcw*, post-conception weeks; *yrs*, years.

**Supplementary Tables**

**Supplementary Table 1. Organisational structure of phenotypes in the GWAS Atlas**. Phenotype organisation of GWAS Atlas by the broader domain level (first column), followed by chapter (second column), subchapter (third column), trait (fourth column), and GWAS level (fifth column), along with the studies PubMed ID (sixth column). *Table is presented as an online file.*

**Supplementary Table 2. Phenotype-associated SNPs permutation test.** Complete results of the permutation test assessing whether the median evolutionary age (third column) of SNPs related to human phenotypes (first column) at the domain/chapter/subchapter/trait level is older (seventh column; positive z-score) or younger (negative z-score) than expected by chance (eight column) given the number of SNPs (second column) and MAF (median MAF in sixth column as reference). The 75th and 25th percentiles of evolutionary age for the SNPs related to each phenotype are also reported (fourth and fifth columns, respectively). *Table is presented as an online file.*

**Supplementary Table 3. Replication of phenotype-associated SNPs permutation test.** Complete results of the permutation test using the EBI Catalog, assessing whether the median evolutionary age (third column) of SNPs related to human phenotypes (first column) is older (seventh column; positive z-score) or younger (negative z-score) than expected by chance (eight column) given the number of SNPs (second column) and MAF (median MAF in sixth column as reference). The 75th and 25th percentiles of evolutionary age for the SNPs related to each phenotype (fourth and fifth columns, respectively), and the PubMed ID of each study are presented (ninth column). *Table is presented as an online file.*

**Supplementary Table 4. Enrichment analysis for signatures of evolutionary forces.** The table presents enrichment scores for GWAS summary statistics of brain, cognitive, and neuropsychiatric phenotypes across evolutionary metrics. The primary focus is on allele age (ARGweave), with additional enrichment results for other evolutionary forces. Asterisks indicate statistically significant enrichment. A brief description of each evolutionary force is provided. *Table is presented as an online file.*

**Supplementary Table 5. Included phenotypes in BRAIN-SNPs.** BRAIN-SNPs are organised in brain structures (first column), combining the SNPs (third column) linked to brain-related phenotypes (second column) from the UK Biobank BIG40 dataset. *Table is presented as an online file.*

**Supplementary Table 6. BRAIN-SNPs permutation test.** Results of the permutation test assessing whether BRAIN-SNPs grouped based on brain structures (first column) display a median evolutionary age (third column) older (fifth column; positive z-score) or younger (negative z-score) than expected by chance (sixth column) given their polygenicity (second column) and MAF (fourth column). Bonferroni *P* value threshold < 5.5 x 10^-3^ (seventh column).

​

| **Brain phenotype** | **N SNPs** | **Median age** | **Median MAF** | **Z-score** | **P value** | **Bonferroni** |
| --- | --- | --- | --- | --- | --- | --- |
| Cortex | 126 | 400170 | 0.223 | -1.87 | 6.0E-02 | 0 |
| Cerebellum | 136 | 619611 | 0.335 | -1.73 | 8.4E-02 | 0 |
| Amygdala | 37 | 332997 | 0.234 | -0.95 | 3.4E-01 | 0 |
| Hippocampus | 29 | 679877 | 0.336 | -0.62 | 5.4E-01 | 0 |
| Pallidum | 20 | 736686 | 0.269 | 0.33 | 7.4E-01 | 0 |
| White matter | 627 | 745857 | 0.300 | 0.49 | 6.2E-01 | 0 |
| Nucleus accumbens | 9 | 1092667 | 0.368 | 0.99 | 3.2E-01 | 0 |
| Thalamus | 17 | 965115 | 0.278 | 1.23 | 2.2E-01 | 0 |
| Caudate nucleus | 22 | 1095415 | 0.288 | 2.18 | 2.9E-02 | 0 |

**Supplementary Table 7. BRAIN-SNPs timeline co-fluctuation.** Spearman’s correlation (third column) and *P* value (fourth column) between the genetic timeline of BRAIN-SNPs associated with different brain structures (first and second columns). Bonferroni *P* value threshold < 1.4 x 10^-3^ (fifth column).

| **Brain structure 1** | **Brain structure 2** | **rho** | **P value** | **Bonferroni** |
| --- | --- | --- | --- | --- |
| Cerebellum | White matter | 0.81 | 5.3E-24 | 1 |
| Cortex | White matter | 0.72 | 3.9E-17 | 1 |
| Cortex | Cerebellum | 0.70 | 7.1E-16 | 1 |
| Cerebellum | Hippocampus | 0.63 | 1.4E-12 | 1 |
| Cerebellum | Amygdala | 0.55 | 2.2E-09 | 1 |
| Cortex | Hippocampus | 0.52 | 3.7E-08 | 1 |
| Hippocampus | White matter | 0.51 | 4.6E-08 | 1 |
| Cortex | Amygdala | 0.51 | 4.9E-08 | 1 |
| Cerebellum | Pallidum | 0.48 | 3.6E-07 | 1 |
| Cerebellum | Caudate nucleus | 0.48 | 4.0E-07 | 1 |
| Hippocampus | Caudate nucleus | 0.48 | 5.8E-07 | 1 |
| Cortex | Pallidum | 0.47 | 6.3E-07 | 1 |
| Amygdala | Hippocampus | 0.45 | 2.2E-06 | 1 |
| White matter | Caudate nucleus | 0.45 | 3.4E-06 | 1 |
| Cerebellum | Nucleus accumbens | 0.43 | 6.8E-06 | 1 |
| Cortex | Caudate nucleus | 0.43 | 7.0E-06 | 1 |
| Amygdala | Caudate nucleus | 0.42 | 1.6E-05 | 1 |
| White matter | Pallidum | 0.41 | 2.8E-05 | 1 |
| Amygdala | White matter | 0.40 | 3.0E-05 | 1 |
| Thalamus | White matter | 0.38 | 1.1E-04 | 1 |
| Amygdala | Nucleus accumbens | 0.37 | 1.4E-04 | 1 |
| Cerebellum | Thalamus | 0.37 | 1.4E-04 | 1 |
| Nucleus accumbens | White matter | 0.36 | 1.9E-04 | 1 |
| Cortex | Nucleus accumbens | 0.36 | 2.1E-04 | 1 |
| Nucleus accumbens | Pallidum | 0.34 | 4.6E-04 | 1 |
| Amygdala | Thalamus | 0.30 | 2.8E-03 | 0 |
| Cortex | Thalamus | 0.29 | 4.0E-03 | 0 |
| Hippocampus | Thalamus | 0.28 | 5.1E-03 | 0 |
| Amygdala | Pallidum | 0.26 | 9.0E-03 | 0 |
| Pallidum | Caudate nucleus | 0.25 | 1.2E-02 | 0 |
| Hippocampus | Pallidum | 0.22 | 2.9E-02 | 0 |
| Thalamus | Pallidum | 0.20 | 4.2E-02 | 0 |
| Hippocampus | Nucleus accumbens | 0.20 | 4.4E-02 | 0 |
| Nucleus accumbens | Caudate nucleus | 0.17 | 8.5E-02 | 0 |
| Thalamus | Caudate nucleus | 0.17 | 8.9E-02 | 0 |
| Thalamus | Nucleus accumbens | 0.01 | 9.3E-01 | 0 |

**Supplementary Table 8. Genes dating estimates.** Genes (first column) are listed with the corresponding chromosome (second column), the number of SNPs within the gene (third column), and the median evolutionary age (fourth column). The 75th and 25th percentiles of evolutionary age for the genes are also provided (fifth and sixth columns, respectively). Additionally, is reported the median quality score (seventh column), which indicates the accuracy of the dating estimates ranging from 0 (low) to 1 (high). For the gene-expression analysis, we highlight the top 10% of genes with the youngest age that also have available expression data from the Allen Human Brain Atlas (eighth column). *Table is presented as an online file.*

**Supplementary Table 9. GWAS included in the present study.** Details of the GWAS included in the gene and gene-set analysis conducted with MAGMA.

| **Phenotype** | **N case** | **N control** | **N total** | **Population** | **Sumstats download link** |
| --- | --- | --- | --- | --- | --- |
| Schizophrenia | 33426 | 54065 | 87491 | EU | https://figshare.com/articles/dataset/cdg2018-bip-scz/14672019 |
| Bipolar disorder | 20129 | 54065 | 74194 | EU | https://figshare.com/articles/dataset/cdg2018-bip-scz/14672019 |
| Autism spectrum disorder | 18381 | 27969 | 46350 | EU | https://figshare.com/articles/dataset/asd2019/14671989 |
| Major depressive disorder | 59851 | 113154 | 173005 | EU | https://figshare.com/articles/dataset/mdd2018/14672085 |
| Alzheimer's disease | 71880 | 383378 | 455258 | EU | https://ctg.cncr.nl/software/summary_statistics |
| Brain volume |  |  | 47316 | EU | https://ctg.cncr.nl/software/summary_statistics |
| Cortical area |  |  | 51665 | EU | https://enigma.ini.usc.edu/research/download-enigma-gwas-results/ |
| Cortical thickness |  |  | 51665 | EU | https://enigma.ini.usc.edu/research/download-enigma-gwas-results/ |
| Intelligence |  |  | 269867 | EU | https://ctg.cncr.nl/software/summary_statistics |
| Sociability |  |  | 452302 | EU | https://www.repository.cam.ac.uk/handle/1810/277812 |

**Supplementary Table 10. Gene-set analysis.** Results of the MAGMA gene-set analysis (third and fourth columns) of the top 10% oldest and youngest genes (first column) testing for enrichment of genes related to brain, cognitive and neuropsychiatric brain phenotypes (second column). Bonferroni *P* value threshold < 2.5 x 10^-3^ (fifth column).

| **Gene-set** | **Phenotype** | **Beta** | **P value** | **Bonferroni** |
| --- | --- | --- | --- | --- |
| Old genes | Alzheimer's disease | -0.05 | 9.8E-01 | 0 |
| Young genes | Alzheimer's disease | 0.05 | 1.1E-02 | 0 |
| Old genes | Autism spectrum disorder | -0.04 | 9.3E-01 | 0 |
| Young genes | Autism spectrum disorder | 0.02 | 1.9E-01 | 0 |
| Old genes | Cortical area | -0.05 | 9.7E-01 | 0 |
| Young genes | Cortical area | 0.08 | 3.5E-04 | 1 |
| Old genes | Bipolar disorder | -0.04 | 9.5E-01 | 0 |
| Young genes | Bipolar disorder | 0.03 | 9.1E-02 | 0 |
| Old genes | Cortical thickness | -0.05 | 9.7E-01 | 0 |
| Young genes | Cortical thickness | 0.03 | 1.0E-01 | 0 |
| Old genes | Brain volume | -0.03 | 7.8E-01 | 0 |
| Young genes | Brain volume | 0.04 | 8.7E-02 | 0 |
| Old genes | Intelligence | -0.11 | 1.0E+00 | 0 |
| Young genes | Intelligence | 0.13 | 1.7E-06 | 1 |
| Old genes | Major depressive disorder | 0.03 | 1.3E-01 | 0 |
| Young genes | Major depressive disorder | 0.02 | 2.2E-01 | 0 |
| Old genes | Schizophrenia | -0.05 | 9.6E-01 | 0 |
| Young genes | Schizophrenia | 0.06 | 1.4E-02 | 0 |
| Old genes | Sociability | -0.04 | 9.4E-01 | 0 |
| Young genes | Sociability | -0.04 | 9.2E-01 | 0 |

**Supplementary Table 11. Normative gene expression analysis.** Normative gene expression levels (extracted from AHBA) of the top 10% genes with the youngest evolutionary age across brain areas using DK atlas (first column). A null random-gene model was applied to assess whether the youngest genes are more expressed (second column; positive z-score) or less expressed (negative z-score) in each brain area than random genes (third column). Bonferroni *P* value threshold < 3 x 10^-3^ (fifth column).

| **Brain region** | **Z-score** | **P value** | **Bonferroni** |
| --- | --- | --- | --- |
| Left pars triangularis | 3.39 | 7.09E-04 | 1 |
| Left posterior cingulate | 2.70 | 6.97E-03 | 0 |
| Left pars opercularis | 2.63 | 8.45E-03 | 0 |
| Left inferior temporal | 2.61 | 8.98E-03 | 0 |
| Left transverse temporal | 2.51 | 1.20E-02 | 0 |
| Left caudal anterior cingulate | 1.96 | 4.98E-02 | 0 |
| Left paracentral | 1.73 | 8.37E-02 | 0 |
| Left rostral middle frontal | 1.69 | 9.09E-02 | 0 |
| Left lingual | 1.67 | 9.55E-02 | 0 |
| Left temporal pole | 1.66 | 9.71E-02 | 0 |
| Left rostral anterior cingulate | 1.13 | 2.59E-01 | 0 |
| Left lateral orbitofrontal | 0.59 | 5.53E-01 | 0 |
| Left superior temporal | 0.49 | 6.22E-01 | 0 |
| Left superior parietal | 0.41 | 6.82E-01 | 0 |
| Left pericalcarine | 0.36 | 7.18E-01 | 0 |
| Left postcentral | 0.33 | 7.42E-01 | 0 |
| Left isthmus cingulate | 0.26 | 7.98E-01 | 0 |
| Left fusiform | 0.14 | 8.87E-01 | 0 |
| Left supramarginal | 0.10 | 9.18E-01 | 0 |
| Left caudal middle frontal | 0.03 | 9.74E-01 | 0 |
| Left precentral | -0.03 | 9.74E-01 | 0 |
| Left medial orbitofrontal | -0.10 | 9.17E-01 | 0 |
| Left precuneus | -0.17 | 8.66E-01 | 0 |
| Left frontal pole | -0.18 | 8.60E-01 | 0 |
| Left superior frontal | -0.35 | 7.30E-01 | 0 |
| Left entorhinal | -0.59 | 5.55E-01 | 0 |
| Left middle temporal | -0.66 | 5.12E-01 | 0 |
| Left parahippocampal | -1.00 | 3.15E-01 | 0 |
| Left insula | -1.17 | 2.44E-01 | 0 |
| Left inferior parietal | -1.19 | 2.33E-01 | 0 |
| Left pars orbitalis | -1.54 | 1.23E-01 | 0 |
| Left cuneus | -1.58 | 1.15E-01 | 0 |
| Left bankssts | -2.89 | 3.83E-03 | 0 |
| Left lateral occipital | -3.27 | 1.08E-03 | 1 |

**Supplementary references**

Abraham A, Labella AL, Benton ML, Rokas A, Capra JA. 2023. GSEL: a fast, flexible python package for detecting signatures of diverse evolutionary forces on genomic regions. Bioinformatics. 39:btad037.

Abraham A, LaBella AL, Capra JA, Rokas A. 2022. Mosaic patterns of selection in genomic regions associated with diverse human traits. PLOS Genet. 18:e1010494.

Autism Spectrum Disorder Working Group of the Psychiatric Genomics Consortium, BUPGEN, Major Depressive Disorder Working Group of the Psychiatric Genomics Consortium, 23andMe Research Team, Grove J, Ripke S, Als TD, Mattheisen M, Walters RK, Won H, Pallesen J, Agerbo E, Andreassen OA, Anney R, Awashti S, Belliveau R, Bettella F, Buxbaum JD, Bybjerg-Grauholm J, Bækvad-Hansen M, Cerrato F, Chambert K, Christensen JH, Churchhouse C, Dellenvall K, Demontis D, De Rubeis S, Devlin B, Djurovic S, Dumont AL, Goldstein JI, Hansen CS, Hauberg ME, Hollegaard MV, Hope S, Howrigan DP, Huang H, Hultman CM, Klei L, Maller J, Martin J, Martin AR, Moran JL, Nyegaard M, Nærland T, Palmer DS, Palotie A, Pedersen CB, Pedersen MG, dPoterba T, Poulsen JB, Pourcain BS, Qvist P, Rehnström K, Reichenberg A, Reichert J, Robinson EB, Roeder K, Roussos P, Saemundsen E, Sandin S, Satterstrom FK, Davey Smith G, Stefansson H, Steinberg S, Stevens CR, Sullivan PF, Turley P, Walters GB, Xu X, Stefansson K, Geschwind DH, Nordentoft M, Hougaard DM, Werge T, Mors O, Mortensen PB, Neale BM, Daly MJ, Børglum AD. 2019. Identification of common genetic risk variants for autism spectrum disorder. Nat Genet. 51:431–444.

Balasubramanian S, Fu Y, Pawashe M, McGillivray P, Jin M, Liu J, Karczewski KJ, MacArthur DG, Gerstein M. 2017. Using ALoFT to determine the impact of putative loss-of-function variants in protein-coding genes. Nat Commun. 8:382.

Barton RA, Harvey PH. 2000. Mosaic evolution of brain structure in mammals. 405.

Buniello A, MacArthur JAL, Cerezo M, Harris LW, Hayhurst J, Malangone C, McMahon A, Morales J, Mountjoy E, Sollis E, Suveges D, Vrousgou O, Whetzel PL, Amode R, Guillen JA, Riat HS, Trevanion SJ, Hall P, Junkins H, Flicek P, Burdett T, Hindorff LA, Cunningham F, Parkinson H. 2019. The NHGRI-EBI GWAS Catalog of published genome-wide association studies, targeted arrays and summary statistics 2019. Nucleic Acids Res. 47:D1005–D1012.

Cammoun L, Gigandet X, Meskaldji D, Thiran JP, Sporns O, Do KQ, Maeder P, Meuli R, Hagmann P. 2012. Mapping the human connectome at multiple scales with diffusion spectrum MRI. J Neurosci Methods. 203:386–397.

Day FR, Ong KK, Perry JRB. 2018. Elucidating the genetic basis of social interaction and isolation. Nat Commun. 9:2457.

De Leeuw CA, Mooij JM, Heskes T, Posthuma D. 2015. MAGMA: Generalized Gene-Set Analysis of GWAS Data. PLOS Comput Biol. 11:e1004219.

Desikan RS, Ségonne F, Fischl B, Quinn BT, Dickerson BC, Blacker D, Buckner RL, Dale AM, Maguire RP, Hyman BT, Albert MS, Killiany RJ. 2006. An automated labeling system for subdividing the human cerebral cortex on MRI scans into gyral based regions of interest. NeuroImage. 31:968–980.

Doan RN, Bae B-I, Cubelos B, Chang C, Hossain AA, Al-Saad S, Mukaddes NM, Oner O, Al-Saffar M, Balkhy S, Gascon GG, Nieto M, Walsh CA. 2016. Mutations in Human Accelerated Regions Disrupt Cognition and Social Behavior. Cell. 167:341-354.e12.

eQTLGen, 23andMe, the Major Depressive Disorder Working Group of the Psychiatric Genomics Consortium, Wray NR, Ripke S, Mattheisen M, Trzaskowski M, Byrne EM, Abdellaoui A, Adams MJ, Agerbo E, Air TM, Andlauer TMF, Bacanu S-A, Bækvad-Hansen M, Beekman AFT, Bigdeli TB, Binder EB, Blackwood DRH, Bryois J, Buttenschøn HN, Bybjerg-Grauholm J, Cai N, Castelao E, Christensen JH, Clarke T-K, Coleman JIR, Colodro-Conde L, Couvy-Duchesne B, Craddock N, Crawford GE, Crowley CA, Dashti HS, Davies G, Deary IJ, Degenhardt F, Derks EM, Direk N, Dolan CV, Dunn EC, Eley TC, Eriksson N, Escott-Price V, Kiadeh FHF, Finucane HK, Forstner AJ, Frank J, Gaspar HA, Gill M, Giusti-Rodríguez P, Goes FS, Gordon SD, Grove J, Hall LS, Hannon E, Hansen CS, Hansen TF, Herms S, Hickie IB, Hoffmann P, Homuth G, Horn C, Hottenga J-J, Hougaard DM, Hu M, Hyde CL, Ising M, Jansen R, Jin F, Jorgenson E, Knowles JA, Kohane IS, Kraft J, Kretzschmar WW, Krogh J, Kutalik Z, Lane JM, Li Y, Li Y, Lind PA, Liu X, Lu L, MacIntyre DJ, MacKinnon DF, Maier RM, Maier W, Marchini J, Mbarek H, McGrath P, McGuffin P, Medland SE, Mehta D, Middeldorp CM, Mihailov E, Milaneschi Y, Milani L, Mill J, Mondimore FM, Montgomery GW, Mostafavi S, Mullins N, Nauck M, Ng B, Nivard MG, Nyholt DR, O’Reilly PF, Oskarsson H, Owen MJ, Painter JN, Pedersen CB, Pedersen MG, Peterson RE, Pettersson E, Peyrot WJ, Pistis G, Posthuma D, Purcell SM, Quiroz JA, Qvist P, Rice JP, Riley BP, Rivera M, Saeed Mirza S, Saxena R, Schoevers R, Schulte EC, Shen L, Shi J, Shyn SI, Sigurdsson E, Sinnamon GBC, Smit JH, Smith DJ, Stefansson H, Steinberg S, Stockmeier CA, Streit F, Strohmaier J, Tansey KE, Teismann H, Teumer A, Thompson W, Thomson PA, Thorgeirsson TE, Tian C, Traylor M, Treutlein J, Trubetskoy V, Uitterlinden AG, Umbricht D, Van Der Auwera S, Van Hemert AM, Viktorin A, Visscher PM, Wang Y, Webb BT, Weinsheimer SM, Wellmann J, Willemsen G, Witt SH, Wu Y, Xi HS, Yang J, Zhang F, Arolt V, Baune BT, Berger K, Boomsma DI, Cichon S, Dannlowski U, De Geus ECJ, DePaulo JR, Domenici E, Domschke K, Esko T, Grabe HJ, Hamilton SP, Hayward C, Heath AC, Hinds DA, Kendler KS, Kloiber S, Lewis G, Li QS, Lucae S, Madden PFA, Magnusson PK, Martin NG, McIntosh AM, Metspalu A, Mors O, Mortensen PB, Müller-Myhsok B, Nordentoft M, Nöthen MM, O’Donovan MC, Paciga SA, Pedersen NL, Penninx BWJH, Perlis RH, Porteous DJ, Potash JB, Preisig M, Rietschel M, Schaefer C, Schulze TG, Smoller JW, Stefansson K, Tiemeier H, Uher R, Völzke H, Weissman MM, Werge T, Winslow AR, Lewis CM, Levinson DF, Breen G, Børglum AD, Sullivan PF. 2018. Genome-wide association analyses identify 44 risk variants and refine the genetic architecture of major depression. Nat Genet. 50:668–681.

Grasby KL, Jahanshad N. 2020. The genetic architecture of the human cerebral cortex.

Grasby KL, Jahanshad N, Painter JN, Colodro-Conde L, Bralten J, Hibar DP, Lind PA, Pizzagalli F, Ching CR, McMahon MAB. 2020. The genetic architecture of the human cerebral cortex. Science. 367:eaay6690.

Hawrylycz MJ, Lein ES, Guillozet-Bongaarts AL, Shen EH, Ng L, Miller JA, Van De Lagemaat LN, Smith KA, Ebbert A, Riley ZL, Abajian C, Beckmann CF, Bernard A, Bertagnolli D, Boe AF, Cartagena PM, Chakravarty MM, Chapin M, Chong J, Dalley RA, Daly BD, Dang C, Datta S, Dee N, Dolbeare TA, Faber V, Feng D, Fowler DR, Goldy J, Gregor BW, Haradon Z, Haynor DR, Hohmann JG, Horvath S, Howard RE, Jeromin A, Jochim JM, Kinnunen M, Lau C, Lazarz ET, Lee C, Lemon TA, Li L, Li Y, Morris JA, Overly CC, Parker PD, Parry SE, Reding M, Royall JJ, Schulkin J, Sequeira PA, Slaughterbeck CR, Smith SC, Sodt AJ, Sunkin SM, Swanson BE, Vawter MP, Williams D, Wohnoutka P, Zielke HR, Geschwind DH, Hof PR, Smith SM, Koch C, Grant SGN, Jones AR. 2012. An anatomically comprehensive atlas of the adult human brain transcriptome. Nature. 489:391–399.

Hibar DP, Stein JL, Renteria ME, Arias-Vasquez A, Desrivières S, Jahanshad N, Toro R, Wittfeld K, Abramovic L, Andersson M. 2015. Common genetic variants influence human subcortical brain structures. Nature. 520:224–229.

Jansen IE, Savage JE, Watanabe K, Bryois J, Williams DM, Steinberg S, Sealock J, Karlsson IK, Hägg S, Athanasiu L, Voyle N, Proitsi P, Witoelar A, Stringer S, Aarsland D, Almdahl IS, Andersen F, Bergh S, Bettella F, Bjornsson S, Brækhus A, Bråthen G, De Leeuw C, Desikan RS, Djurovic S, Dumitrescu L, Fladby T, Hohman TJ, Jonsson PV, Kiddle SJ, Rongve A, Saltvedt I, Sando SB, Selbæk G, Shoai M, Skene NG, Snaedal J, Stordal E, Ulstein ID, Wang Y, White LR, Hardy J, Hjerling-Leffler J, Sullivan PF, Van Der Flier WM, Dobson R, Davis LK, Stefansson H, Stefansson K, Pedersen NL, Ripke S, Andreassen OA, Posthuma D. 2019. Genome-wide meta-analysis identifies new loci and functional pathways influencing Alzheimer’s disease risk. Nat Genet. 51:404–413.

Jansen PR, Nagel M, Watanabe K, Wei Y, Savage JE, De Leeuw CA, Van Den Heuvel MP, Van Der Sluis S, Posthuma D. 2020. Genome-wide meta-analysis of brain volume identifies genomic loci and genes shared with intelligence. Nat Commun. 11:5606.

Miller JA, Ding S-L, Sunkin SM, Smith KA, Ng L, Szafer A, Ebbert A, Riley ZL, Royall JJ, Aiona K, Arnold JM, Bennet C, Bertagnolli D, Brouner K, Butler S, Caldejon S, Carey A, Cuhaciyan C, Dalley RA, Dee N, Dolbeare TA, Facer BAC, Feng D, Fliss TP, Gee G, Goldy J, Gourley L, Gregor BW, Gu G, Howard RE, Jochim JM, Kuan CL, Lau C, Lee C-K, Lee F, Lemon TA, Lesnar P, McMurray B, Mastan N, Mosqueda N, Naluai-Cecchini T, Ngo N-K, Nyhus J, Oldre A, Olson E, Parente J, Parker PD, Parry SE, Stevens A, Pletikos M, Reding M, Roll K, Sandman D, Sarreal M, Shapouri S, Shapovalova NV, Shen EH, Sjoquist N, Slaughterbeck CR, Smith M, Sodt AJ, Williams D, Zöllei L, Fischl B, Gerstein MB, Geschwind DH, Glass IA, Hawrylycz MJ, Hevner RF, Huang H, Jones AR, Knowles JA, Levitt P, Phillips JW, Šestan N, Wohnoutka P, Dang C, Bernard A, Hohmann JG, Lein ES. 2014. Transcriptional landscape of the prenatal human brain. Nature. 508:199–206.

Nurk S, Koren S, Rhie A, Rautiainen M, Bzikadze AV, Mikheenko A, Vollger MR, Altemose N, Uralsky L, Gershman A, Aganezov S, Hoyt SJ, Diekhans M, Logsdon GA, Alonge M, Antonarakis SE, Borchers M, Bouffard GG, Brooks SY, Caldas GV, Chen N-C, Cheng H, Chin C-S, Chow W, de Lima LG, Dishuck PC, Durbin R, Dvorkina T, Fiddes IT, Formenti G, Fulton RS, Fungtammasan A, Garrison E, Grady PGS, Graves-Lindsay TA, Hall IM, Hansen NF, Hartley GA, Haukness M, Howe K, Hunkapiller MW, Jain C, Jain M, Jarvis ED, Kerpedjiev P, Kirsche M, Kolmogorov M, Korlach J, Kremitzki M, Li H, Maduro VV, Marschall T, McCartney AM, McDaniel J, Miller DE, Mullikin JC, Myers EW, Olson ND, Paten B, Peluso P, Pevzner PA, Porubsky D, Potapova T, Rogaev EI, Rosenfeld JA, Salzberg SL, Schneider VA, Sedlazeck FJ, Shafin K, Shew CJ, Shumate A, Sims Y, Smit AFA, Soto DC, Sovic I, Storer JM, Streets A, Sullivan BA, Thibaud-Nissen F, Torrance J, Wagner J, Walenz BP, Wenger A, Wood JMD, Xiao C, Yan SM, Young AC, Zarate S, Surti U, McCoy RC, Dennis MY, Alexandrov IA, Gerton JL, Schatz MC, Eichler EE, Miga KH, Phillippy AM. 2022. The complete sequence of a human genome.

Pijnenburg R, Scholtens LH, Ardesch DJ, De Lange SC, Wei Y, Van Den Heuvel MP. 2021. Myelo- and cytoarchitectonic microstructural and functional human cortical atlases reconstructed in common MRI space. NeuroImage. 239:118274.

Pollard KS, Salama SR, Lambert N, Lambot M-A, Coppens S, Pedersen JS, Katzman S, King B, Onodera C, Siepel A, Kern AD, Dehay C, Igel H, Ares M, Vanderhaeghen P, Haussler D. 2006. An RNA gene expressed during cortical development evolved rapidly in humans. Nature. 443:167–172.

Purcell S, Neale B, Todd-Brown K, Thomas L, Ferreira MAR, Bender D, Maller J, Sklar P, De Bakker PIW, Daly MJ, Sham PC. 2007. PLINK: A Tool Set for Whole-Genome Association and Population-Based Linkage Analyses. Am J Hum Genet. 81:559–575.

Rasmussen MD, Hubisz MJ, Gronau I, Siepel A. 2014. Genome-Wide Inference of Ancestral Recombination Graphs. PLoS Genet. 10:e1004342.

Ruderfer DM, Ripke S, McQuillin A, Boocock J, Stahl EA, Pavlides JMW, Mullins N, Charney AW, Ori APS, Loohuis LMO, Domenici E, Di Florio A, Papiol S, Kalman JL, Trubetskoy V, Adolfsson R, Agartz I, Agerbo E, Akil H, Albani D, Albus M, Alda M, Alexander M, Alliey-Rodriguez N, Als TD, Amin F, Anjorin A, Arranz MJ, Awasthi S, Bacanu SA, Badner JA, Baekvad-Hansen M, Bakker S, Band G, Barchas JD, Barroso I, Bass N, Bauer M, Baune BT, Begemann M, Bellenguez C, Belliveau RA, Bellivier F, Bender S, Bene J, Bergen SE, Berrettini WH, Bevilacqua E, Biernacka JM, Bigdeli TB, Black DW, Blackburn H, Blackwell JM, Blackwood DHR, Pedersen CB, Boehnke M, Boks M, Borglum AD, Bramon E, Breen G, Brown MA, Bruggeman R, Buccola NG, Buckner RL, Budde M, Bulik-Sullivan B, Bumpstead SJ, Bunney W, Burmeister M, Buxbaum JD, Bybjerg-Grauholm J, Byerley W, Cahn W, Cai G, Cairns MJ, Campion D, Cantor RM, Carr VJ, Carrera N, Casas JP, Casas M, Catts SV, Cervantes P, Chambert KD, Chan RCK, Chen EYH, Chen RYL, Cheng W, Cheung EFC, Chong SA, Clarke T-K, Cloninger CR, Cohen D, Cohen N, Coleman JRI, Collier DA, Cormican P, Coryell W, Craddock N, Craig DW, Crespo-Facorro B, Crowley JJ, Cruceanu C, Curtis D, Czerski PM, Dale AM, Daly MJ, Dannlowski U, Darvasi A, Davidson M, Davis KL, De Leeuw CA, Degenhardt F, Del Favero J, DeLisi LE, Deloukas P, Demontis D, DePaulo JR, Di Forti M, Dikeos D, Dinan T, Djurovic S, Dobbyn AL, Donnelly P, Donohoe G, Drapeau E, Dronov S, Duan J, Dudbridge F, Duncanson A, Edenberg H, Edkins S, Ehrenreich H, Eichhammer P, Elvsashagen T, Eriksson J, Escott-Price V, Esko T, Essioux L, Etain B, Fan CC, Farh K-H, Farrell MS, Flickinger M, Foroud TM, Forty L, Frank J, Franke L, Fraser C, Freedman R, Freeman C, Freimer NB, Friedman JI, Fromer M, Frye MA, Fullerton JM, Gade K, Garnham J, Gaspar HA, Gejman PV, Genovese G, Georgieva L, Giambartolomei C, Giannoulatou E, Giegling I, Gill M, Gillman M, Pedersen MG, Giusti-Rodriguez P, Godard S, Goes F, Goldstein JI, Gopal S, Gordon SD, Gordon-Smith K, Gratten J, Gray E, Green EK, Green MJ, Greenwood TA, Grigoroiu-Serbanescu M, Grove J, Guan W, Gurling H, Parra JG, Gwilliam R, De Haan L, Hall J, Hall M-H, Hammer C, Hammond N, Hamshere ML, Hansen M, Hansen T, Haroutunian V, Hartmann AM, Hauser J, Hautzinger M, Heilbronner U, Hellenthal G, Henskens FA, Herms S, Hipolito M, Hirschhorn JN, Hoffmann P, Hollegaard MV, Hougaard DM, Huang H, Huckins L, Hultman CM, Hunt SE, Ikeda M, Iwata N, Iyegbe C, Jablensky AV, Jamain S, Jankowski J, Jayakumar A, Joa I, Jones I, Jones LA, Jonsson EG, Julia A, Jureus A, Kahler AK, Kahn RS, Kalaydjieva L, Kandaswamy R, Karachanak-Yankova S, Karjalainen J, Karlsson R, Kavanagh D, Keller MC, Kelly BJ, Kelsoe J, Kennedy JL, Khrunin A, Kim Y, Kirov G, Kittel-Schneider S, Klovins J, Knight J, Knott SV, Knowles JA, Kogevinas M, Konte B, Kravariti E, Kucinskas V, Kucinskiene ZA, Kupka R, Kuzelova-Ptackova H, Landen M, Langford C, Laurent C, Lawrence J, Lawrie S, Lawson WB, Leber M, Leboyer M, Lee PH, Keong JLC, Legge SE, Lencz T, Lerer B, Levinson DF, Levy SE, Lewis CM, Li JZ, Li M, Li QS, Li T, Liang K-Y, Liddle J, Lieberman J, Limborska S, Lin K, Linszen DH, Lissowska J, Liu C, Liu J, Lonnqvist J, Loughland CM, Lubinski J, Lucae S, Macek M, MacIntyre DJ, Magnusson PKE, Maher BS, Mahon PB, Maier W, Malhotra AK, Mallet J, Malt UF, Markus HS, Marsal S, Martin NG, Mata I, Mathew CG, Mattheisen M, Mattingsdal M, Mayoral F, McCann OT, McCarley RW, McCarroll SA, McCarthy MI, McDonald C, McElroy SL, McGuffin P, McInnis MG, McIntosh AM, McKay JD, McMahon FJ, Medeiros H, Medland SE, Meier S, Meijer CJ, Melegh B, Melle I, Meng F, Mesholam-Gately RI, Metspalu A, Michie PT, Milani L, Milanova V, Mitchell PB, Mokrab Y, Montgomery GW, Moran JL, Morken G, Morris DW, Mors O, Mortensen PB, Mowry BJ, Mühleisen TW, Müller-Myhsok B, Murphy KC, Murray RM, Myers RM, Myin-Germeys I, Neale BM, Nelis M, Nenadic I, Nertney DA, Nestadt G, Nicodemus KK, Nievergelt CM, Nikitina-Zake L, Nimgaonkar V, Nisenbaum L, Nordentoft M, Nordin A, Nöthen MM, Nwulia EA, O’Callaghan E, O’Donovan C, O’Dushlaine C, O’Neill FA, Oedegaard KJ, Oh S-Y, Olincy A, Olsen L, Oruc L, Van Os J, Owen MJ, Paciga SA, Palmer CNA, Palotie A, Pantelis C, Papadimitriou GN, Parkhomenko E, Pato C, Pato MT, Paunio T, Pearson R, Perkins DO, Perlis RH, Perry A, Pers TH, Petryshen TL, Pfennig A, Picchioni M, Pietilainen O, Pimm J, Pirinen M, Plomin R, Pocklington AJ, Posthuma D, Potash JB, Potter SC, Powell J, Price A, Pulver AE, Purcell SM, Quested D, Ramos-Quiroga JA, Rasmussen HB, Rautanen A, Ravindrarajah R, Regeer EJ, Reichenberg A, Reif A, Reimers MA, Ribases M, Rice JP, Richards AL, Ricketts M, Riley BP, Rivas F, Rivera M, Roffman JL, Rouleau GA, Roussos P, Rujescu D, Salomaa V, Sanchez-Mora C, Sanders AR, Sawcer SJ, Schall U, Schatzberg AF, Scheftner WA, Schofield PR, Schork NJ, Schwab SG, Scolnick EM, Scott LJ, Scott RJ, Seidman LJ, Serretti A, Sham PC, Weickert CS, Shehktman T, Shi J, Shilling PD, Sigurdsson E, Silverman JM, Sim K, Slaney C, Slominsky P, Smeland OB, Smoller JW, So H-C, Sobell JL, Soderman E, Hansen CS, Spencer CCA, Spijker AT, St Clair D, Stefansson H, Stefansson K, Steinberg S, Stogmann E, Stordal E, Strange A, Straub RE, Strauss JS, Streit F, Strengman E, Strohmaier J, Stroup TS, Su Z, Subramaniam M, Suvisaari J, Svrakic DM, Szatkiewicz JP, Szelinger S, Tashakkori-Ghanbaria A, Thirumalai S, Thompson RC, Thorgeirsson TE, Toncheva D, Tooney PA, Tosato S, Toulopoulou T, Trembath RC, Treutlein J, Trubetskoy V, Turecki G, Vaaler AE, Vedder H, Vieta E, Vincent J, Visscher PM, Viswanathan AC, Vukcevic D, Waddington J, Waller M, Walsh D, Walshe M, Walters JTR, Wang D, Wang Q, Wang W, Wang Y, Watson SJ, Webb BT, Weickert TW, Weinberger DR, Weisbrod M, Weiser M, Werge T, Weston P, Whittaker P, Widaa S, Wiersma D, Wildenauer DB, Williams NM, Williams S, Witt SH, Wolen AR, Wong EHM, Wood NW, Wormley BK, Wu JQ, Xi S, Xu W, Young AH, Zai CC, Zandi P, Zhang P, Zheng X, Zimprich F, Zollner S, Corvin A, Fanous AH, Cichon S, Rietschel M, Gershon ES, Schulze TG, Cuellar-Barboza AB, Forstner AJ, Holmans PA, Nurnberger JI, Andreassen OA, Lee SH, O’Donovan MC, Sullivan PF, Ophoff RA, Wray NR, Sklar P, Kendler KS. 2018. Genomic Dissection of Bipolar Disorder and Schizophrenia, Including 28 Subphenotypes. Cell. 173:1705-1715.e16.

Savage JE, Jansen PR, Stringer S, Watanabe K, Bryois J, De Leeuw CA, Nagel M, Awasthi S, Barr PB, Coleman JRI, Grasby KL, Hammerschlag AR, Kaminski JA, Karlsson R, Krapohl E, Lam M, Nygaard M, Reynolds CA, Trampush JW, Young H, Zabaneh D, Hägg S, Hansell NK, Karlsson IK, Linnarsson S, Montgomery GW, Muñoz-Manchado AB, Quinlan EB, Schumann G, Skene NG, Webb BT, White T, Arking DE, Avramopoulos D, Bilder RM, Bitsios P, Burdick KE, Cannon TD, Chiba-Falek O, Christoforou A, Cirulli ET, Congdon E, Corvin A, Davies G, Deary IJ, DeRosse P, Dickinson D, Djurovic S, Donohoe G, Conley ED, Eriksson JG, Espeseth T, Freimer NA, Giakoumaki S, Giegling I, Gill M, Glahn DC, Hariri AR, Hatzimanolis A, Keller MC, Knowles E, Koltai D, Konte B, Lahti J, Le Hellard S, Lencz T, Liewald DC, London E, Lundervold AJ, Malhotra AK, Melle I, Morris D, Need AC, Ollier W, Palotie A, Payton A, Pendleton N, Poldrack RA, Räikkönen K, Reinvang I, Roussos P, Rujescu D, Sabb FW, Scult MA, Smeland OB, Smyrnis N, Starr JM, Steen VM, Stefanis NC, Straub RE, Sundet K, Tiemeier H, Voineskos AN, Weinberger DR, Widen E, Yu J, Abecasis G, Andreassen OA, Breen G, Christiansen L, Debrabant B, Dick DM, Heinz A, Hjerling-Leffler J, Ikram MA, Kendler KS, Martin NG, Medland SE, Pedersen NL, Plomin R, Polderman TJC, Ripke S, Van Der Sluis S, Sullivan PF, Vrieze SI, Wright MJ, Posthuma D. 2018. Genome-wide association meta-analysis in 269,867 individuals identifies new genetic and functional links to intelligence. Nat Genet. 50:912–919.

Smith SM, Douaud G, Chen W, Hanayik T, Alfaro-Almagro F, Sharp K, Elliott LT. 2021. An expanded set of genome-wide association studies of brain imaging phenotypes in UK Biobank. Nat Neurosci. 24:737–745.

The 1000 Genomes Project Consortium, Corresponding authors, Auton A, Abecasis GR, Steering committee, Altshuler DM, Durbin RM, Abecasis GR, Bentley DR, Chakravarti A, Clark AG, Donnelly P, Eichler EE, Flicek P, Gabriel SB, Gibbs RA, Green ED, Hurles ME, Knoppers BM, Korbel JO, Lander ES, Lee C, Lehrach H, Mardis ER, Marth GT, McVean GA, Nickerson DA, Schmidt JP, Sherry ST, Wang J, Wilson RK, Production group, Baylor College of Medicine, Gibbs RA, Boerwinkle E, Doddapaneni H, Han Y, Korchina V, Kovar C, Lee S, Muzny D, Reid JG, Zhu Y, BGI-Shenzhen, Wang J, Chang Y, Feng Q, Fang X, Guo X, Jian M, Jiang H, Jin X, Lan T, Li G, Li J, Li Y, Liu S, Liu X, Lu Y, Ma X, Tang M, Wang B, Wang G, Wu H, Wu R, Xu X, Yin Y, Zhang D, Zhang W, Zhao J, Zhao M, Zheng X, Broad Institute of MIT and Harvard, Lander ES, Altshuler DM, Gabriel SB, Gupta N, Coriell Institute for Medical Research, Gharani N, Toji LH, Gerry NP, Resch AM, European Molecular Biology Laboratory, European Bioinformatics Institute, Flicek P, Barker J, Clarke L, Gil L, Hunt SE, Kelman G, Kulesha E, Leinonen R, McLaren WM, Radhakrishnan R, Roa A, Smirnov D, Smith RE, Streeter I, Thormann A, Toneva I, Vaughan B, Zheng-Bradley X, Illumina, Bentley DR, Grocock R, Humphray S, James T, Kingsbury Z, Max Planck Institute for Molecular Genetics, Lehrach H, Sudbrak R, Albrecht MW, Amstislavskiy VS, Borodina TA, Lienhard M, Mertes F, Sultan M, Timmermann B, Yaspo M-L, McDonnell Genome Institute at Washington University, Mardis ER, Wilson RK, Fulton L, Fulton R, US National Institutes of Health, Sherry ST, Ananiev V, Belaia Z, Beloslyudtsev D, Bouk N, Chen C, Church D, Cohen R, Cook C, Garner J, Hefferon T, Kimelman M, Liu C, Lopez J, Meric P, O’Sullivan C, Ostapchuk Y, Phan L, Ponomarov S, Schneider V, Shekhtman E, Sirotkin K, Slotta D, Zhang H, University of Oxford, McVean GA, Wellcome Trust Sanger Institute, Durbin RM, Balasubramaniam S, Burton J, Danecek P, Keane TM, Kolb-Kokocinski A, McCarthy S, Stalker J, Quail M, Analysis group, Affymetrix, Schmidt JP, Davies CJ, Gollub J, Webster T, Wong B, Zhan Y, Albert Einstein College of Medicine, Auton A, Campbell CL, Kong Y, Marcketta A, Baylor College of Medicine, Gibbs RA, Yu F, Antunes L, Bainbridge M, Muzny D, Sabo A, Huang Z, BGI-Shenzhen, Wang J, Coin LJM, Fang L, Guo X, Jin X, Li G, Li Q, Li Y, Li Z, Lin H, Liu B, Luo R, Shao H, Xie Y, Ye C, Yu C, Zhang F, Zheng H, Zhu H, Bilkent University, Alkan C, Dal E, Kahveci F, Boston College, Marth GT, Garrison EP, Kural D, Lee W-P, Fung Leong W, Stromberg M, Ward AN, Wu J, Zhang M, Broad Institute of MIT and Harvard, Daly MJ, DePristo MA, Handsaker RE, Altshuler DM, Banks E, Bhatia G, Del Angel G, Gabriel SB, Genovese G, Gupta N, Li H, Kashin S, Lander ES, McCarroll SA, Nemesh JC, Poplin RE, Cold Spring Harbor Laboratory, Yoon SC, Lihm J, Makarov V, Cornell University, Clark AG, Gottipati S, Keinan A, Rodriguez-Flores JL, European Molecular Biology Laboratory, Korbel JO, Rausch T, Fritz MH, Stütz AM, European Molecular Biology Laboratory, European Bioinformatics Institute, Flicek P, Beal K, Clarke L, Datta A, Herrero J, McLaren WM, Ritchie GRS, Smith RE, Zerbino D, Zheng-Bradley X, Harvard University, Sabeti PC, Shlyakhter I, Schaffner SF, Vitti J, Human Gene Mutation Database, Cooper DN, Ball EV, Stenson PD, Illumina, Bentley DR, Barnes B, Bauer M, Keira Cheetham R, Cox A, Eberle M, Humphray S, Kahn S, Murray L, Peden J, Shaw R, Icahn School of Medicine at Mount Sinai, Kenny EE, Louisiana State University, Batzer MA, Konkel MK, Walker JA, Massachusetts General Hospital, MacArthur DG, Lek M, Max Planck Institute for Molecular Genetics, Sudbrak R, Amstislavskiy VS, Herwig R, McDonnell Genome Institute at Washington University, Mardis ER, Ding L, Koboldt DC, Larson D, Ye K, McGill University, Gravel S, National Eye Institute, NIH, Swaroop A, Chew E, New York Genome Center, Lappalainen T, Erlich Y, Gymrek M, Frederick Willems T, Ontario Institute for Cancer Research, Simpson JT, Pennsylvania State University, Shriver MD, Rutgers Cancer Institute of New Jersey, Rosenfeld JA, Stanford University, Bustamante CD, Montgomery SB, De La Vega FM, Byrnes JK, Carroll AW, DeGorter MK, Lacroute P, Maples BK, Martin AR, Moreno-Estrada A, Shringarpure SS, Zakharia F, Tel-Aviv University, Halperin E, Baran Y, The Jackson Laboratory for Genomic Medicine, Lee C, Cerveira E, Hwang J, Malhotra A, Plewczynski D, Radew K, Romanovitch M, Zhang C, Thermo Fisher Scientific, Hyland FCL, Translational Genomics Research Institute, Craig DW, Christoforides A, Homer N, Izatt T, Kurdoglu AA, Sinari SA, Squire K, US National Institutes of Health, Sherry ST, Xiao C, University of California, San Diego, Sebat J, Antaki D, Gujral M, Noor A, Ye K, University of California, San Francisco, Burchard EG, Hernandez RD, Gignoux CR, University of California, Santa Cruz, Haussler D, Katzman SJ, James Kent W, University of Chicago, Howie B, University College London, Ruiz-Linares A, University of Geneva, Dermitzakis ET, University of Maryland School of Medicine, Devine SE, University of Michigan, Abecasis GR, Min Kang H, Kidd JM, Blackwell T, Caron S, Chen W, Emery S, Fritsche L, Fuchsberger C, Jun G, Li B, Lyons R, Scheller C, Sidore C, Song S, Sliwerska E, Taliun D, Tan A, Welch R, Kate Wing M, Zhan X, University of Montréal, Awadalla P, Hodgkinson A, University of North Carolina at Chapel Hill, Li Y, University of North Carolina at Charlotte, Shi X, Quitadamo A, University of Oxford, Lunter G, McVean GA, Marchini JL, Myers S, Churchhouse C, Delaneau O, Gupta-Hinch A, Kretzschmar W, Iqbal Z, Mathieson I, Menelaou A, Rimmer A, Xifara DK, University of Puerto Rico, Oleksyk TK, University of Texas Health Sciences Center at Houston, Fu Y, Liu X, Xiong M, University of Utah, Jorde L, Witherspoon D, Xing J, University of Washington, Eichler EE, Browning BL, Browning SR, Hormozdiari F, Sudmant PH, Weill Cornell Medical College, Khurana E, Wellcome Trust Sanger Institute, Durbin RM, Hurles ME, Tyler-Smith C, Albers CA, Ayub Q, Balasubramaniam S, Chen Y, Colonna V, Danecek P, Jostins L, Keane TM, McCarthy S, Walter K, Xue Y, Yale University, Gerstein MB, Abyzov A, Balasubramanian S, Chen J, Clarke D, Fu Y, Harmanci AO, Jin M, Lee D, Liu J, Jasmine Mu X, Zhang J, Zhang Y, Structural variation group, BGI-Shenzhen, Li Y, Luo R, Zhu H, Bilkent University, Alkan C, Dal E, Kahveci F, Boston College, Marth GT, Garrison EP, Kural D, Lee W-P, Ward AN, Wu J, Zhang M, Broad Institute of MIT and Harvard, McCarroll SA, Handsaker RE, Altshuler DM, Banks E, Del Angel G, Genovese G, Hartl C, Li H, Kashin S, Nemesh JC, Shakir K, Cold Spring Harbor Laboratory, Yoon SC, Lihm J, Makarov V, Cornell University, Degenhardt J, European Molecular Biology Laboratory, Korbel JO, Fritz MH, Meiers S, Raeder B, Rausch T, Stütz AM, European Molecular Biology Laboratory, European Bioinformatics Institute, Flicek P, Paolo Casale F, Clarke L, Smith RE, Stegle O, Zheng-Bradley X, Illumina, Bentley DR, Barnes B, Keira Cheetham R, Eberle M, Humphray S, Kahn S, Murray L, Shaw R, Leiden University Medical Center, Lameijer E-W, Louisiana State University, Batzer MA, Konkel MK, Walker JA, McDonnell Genome Institute at Washington University, Ding L, Hall I, Ye K, Stanford University, Lacroute P, The Jackson Laboratory for Genomic Medicine, Lee C, Cerveira E, Malhotra A, Hwang J, Plewczynski D, Radew K, Romanovitch M, Zhang C, Translational Genomics Research Institute, Craig DW, Homer N, US National Institutes of Health, Church D, Xiao C, University of California, San Diego, Sebat J, Antaki D, Bafna V, Michaelson J, Ye K, University of Maryland School of Medicine, Devine SE, Gardner EJ, University of Michigan, Abecasis GR, Kidd JM, Mills RE, Dayama G, Emery S, Jun G, University of North Carolina at Charlotte, Shi X, Quitadamo A, University of Oxford, Lunter G, McVean GA, University of Texas MD Anderson Cancer Center, Chen K, Fan X, Chong Z, Chen T, University of Utah, Witherspoon D, Xing J, University of Washington, Eichler EE, Chaisson MJ, Hormozdiari F, Huddleston J, Malig M, Nelson BJ, Sudmant PH, Vanderbilt University School of Medicine, Parrish NF, Weill Cornell Medical College, Khurana E, Wellcome Trust Sanger Institute, Hurles ME, Blackburne B, Lindsay SJ, Ning Z, Walter K, Zhang Y, Yale University, Gerstein MB, Abyzov A, Chen J, Clarke D, Lam H, Jasmine Mu X, Sisu C, Zhang J, Zhang Y, Exome group, Baylor College of Medicine, Gibbs RA, Yu F, Bainbridge M, Challis D, Evani US, Kovar C, Lu J, Muzny D, Nagaswamy U, Reid JG, Sabo A, Yu J, BGI-Shenzhen, Guo X, Li W, Li Y, Wu R, Boston College, Marth GT, Garrison EP, Fung Leong W, Ward AN, Broad Institute of MIT and Harvard, Del Angel G, DePristo MA, Gabriel SB, Gupta N, Hartl C, Poplin RE, Cornell University, Clark AG, Rodriguez-Flores JL, European Molecular Biology Laboratory, European Bioinformatics Institute, Flicek P, Clarke L, Smith RE, Zheng-Bradley X, Massachusetts General Hospital, MacArthur DG, McDonnell Genome Institute at Washington University, Mardis ER, Fulton R, Koboldt DC, McGill University, Gravel S, Stanford University, Bustamante CD, Translational Genomics Research Institute, Craig DW, Christoforides A, Homer N, Izatt T, US National Institutes of Health, Sherry ST, Xiao C, University of Geneva, Dermitzakis ET, University of Michigan, Abecasis GR, Min Kang H, University of Oxford, McVean GA, Yale University, Gerstein MB, Balasubramanian S, Habegger L, Functional interpretation group, Cornell University, Yu H, European Molecular Biology Laboratory, European Bioinformatics Institute, Flicek P, Clarke L, Cunningham F, Dunham I, Zerbino D, Zheng-Bradley X, Harvard University, Lage K, Berg Jespersen J, Horn H, Stanford University, Montgomery SB, DeGorter MK, Weill Cornell Medical College, Khurana E, Wellcome Trust Sanger Institute, Tyler-Smith C, Chen Y, Colonna V, Xue Y, Yale University, Gerstein MB, Balasubramanian S, Fu Y, Kim D, Chromosome Y group, Albert Einstein College of Medicine, Auton A, Marcketta A, American Museum of Natural History, Desalle R, Narechania A, Arizona State University, Wilson Sayres MA, Boston College, Garrison EP, Broad Institute of MIT and Harvard, Handsaker RE, Kashin S, McCarroll SA, Cornell University, Rodriguez-Flores JL, European Molecular Biology Laboratory, European Bioinformatics Institute, Flicek P, Clarke L, Zheng-Bradley X, New York Genome Center, Erlich Y, Gymrek M, Frederick Willems T, Stanford University, Bustamante CD, Mendez FL, David Poznik G, Underhill PA, The Jackson Laboratory for Genomic Medicine, Lee C, Cerveira E, Malhotra A, Romanovitch M, Zhang C, University of Michigan, Abecasis GR, University of Queensland, Coin L, Shao H, Virginia Bioinformatics Institute, Mittelman D, Wellcome Trust Sanger Institute, Tyler-Smith C, Ayub Q, Banerjee R, Cerezo M, Chen Y, Fitzgerald TW, Louzada S, Massaia A, McCarthy S, Ritchie GR, Xue Y, Yang F, Data coordination center group, Baylor College of Medicine, Gibbs RA, Kovar C, Kalra D, Hale W, Muzny D, Reid JG, BGI-Shenzhen, Wang J, Dan X, Guo X, Li G, Li Y, Ye C, Zheng X, Broad Institute of MIT and Harvard, Altshuler DM, European Molecular Biology Laboratory, European Bioinformatics Institute, Flicek P, Clarke L, Zheng-Bradley X, Illumina, Bentley DR, Cox A, Humphray S, Kahn S, Max Planck Institute for Molecular Genetics, Sudbrak R, Albrecht MW, Lienhard M, McDonnell Genome Institute at Washington University, Larson D, Translational Genomics Research Institute, Craig DW, Izatt T, Kurdoglu AA, US National Institutes of Health, Sherry ST, Xiao C, University of California, Santa Cruz, Haussler D, University of Michigan, Abecasis GR, University of Oxford, McVean GA, Wellcome Trust Sanger Institute, Durbin RM, Balasubramaniam S, Keane TM, McCarthy S, Stalker J, Samples and ELSI group, Chakravarti A, Knoppers BM, Abecasis GR, Barnes KC, Beiswanger C, Burchard EG, Bustamante CD, Cai H, Cao H, Durbin RM, Gerry NP, Gharani N, Gibbs RA, Gignoux CR, Gravel S, Henn B, Jones D, Jorde L, Kaye JS, Keinan A, Kent A, Kerasidou A, Li Y, Mathias R, McVean GA, Moreno-Estrada A, Ossorio PN, Parker M, Resch AM, Rotimi CN, Royal CD, Sandoval K, Su Y, Sudbrak R, Tian Z, Tishkoff S, Toji LH, Tyler-Smith C, Via M, Wang Y, Yang H, Yang L, Zhu J, Sample collection, British from England and Scotland (GBR), Bodmer W, Colombians in Medellín, Colombia (CLM), Bedoya G, Ruiz-Linares A, Han Chinese South (CHS), Cai Z, Gao Y, Chu J, Finnish in Finland (FIN), Peltonen L, Iberian Populations in Spain (IBS), Garcia-Montero A, Orfao A, Puerto Ricans in Puerto Rico (PUR), Dutil J, Martinez-Cruzado JC, Oleksyk TK, African Caribbean in Barbados (ACB), Barnes KC, Mathias RA, Hennis A, Watson H, McKenzie C, Bengali in Bangladesh (BEB), Qadri F, LaRocque R, Sabeti PC, Chinese Dai in Xishuangbanna, China (CDX), Zhu J, Deng X, Esan in Nigeria (ESN), Sabeti PC, Asogun D, Folarin O, Happi C, Omoniwa O, Stremlau M, Tariyal R, Gambian in Western Division – Mandinka (GWD), Jallow M, Sisay Joof F, Corrah T, Rockett K, Kwiatkowski D, Indian Telugu in the UK (ITU) and Sri Lankan Tamil in the UK (STU), Kooner J, Kinh in Ho Chi Minh City, Vietnam (KHV), Tịnh Hiê`n T, Dunstan SJ, Thuy Hang N, Mende in Sierra Leone (MSL), Fonnie R, Garry R, Kanneh L, Moses L, Sabeti PC, Schieffelin J, Grant DS, Peruvian in Lima, Peru (PEL), Gallo C, Poletti G, Punjabi in Lahore, Pakistan (PJL), Saleheen D, Rasheed A, Scientific management, Brooks LD, Felsenfeld AL, McEwen JE, Vaydylevich Y, Green ED, Duncanson A, Dunn M, Schloss JA, Wang J, Yang H, Writing group, Auton A, Brooks LD, Durbin RM, Garrison EP, Min Kang H, Korbel JO, Marchini JL, McCarthy S, McVean GA, Abecasis GR. 2015. A global reference for human genetic variation. Nature. 526:68–74.

the Haplotype Reference Consortium. 2016. A reference panel of 64,976 haplotypes for genotype imputation. Nat Genet. 48:1279–1283.

Tissink E, De Lange SC, Savage JE, Wightman DP, De Leeuw CA, Kelly KM, Nagel M, Van Den Heuvel MP, Posthuma D. 2022. Genome-wide association study of cerebellar volume provides insights into heritable mechanisms underlying brain development and mental health. Commun Biol. 5:710.

Tuller T, Kupiec M, Ruppin E. 2008. Evolutionary Rate and Gene Expression Across Different Brain Regions. Genome Biol. 9:R142.

Watanabe K, Stringer S, Frei O, Umićević Mirkov M, De Leeuw C, Polderman TJC, Van Der Sluis S, Andreassen OA, Neale BM, Posthuma D. 2019. A global overview of pleiotropy and genetic architecture in complex traits. Nat Genet. 51:1339–1348.

Watanabe K, Taskesen E, Van Bochoven A, Posthuma D. 2017. Functional mapping and annotation of genetic associations with FUMA. Nat Commun. 8:1826.

Wei Y, Lange SC, Pijnenburg R, Scholtens LH, Ardesch DJ, Watanabe K, Posthuma D, Heuvel MP. 2022. Statistical testing in transcriptomic‐neuroimaging studies: A how‐to and evaluation of methods assessing spatial and gene specificity. Hum Brain Mapp. 43:885–901.

Whiting BA, Barton RA. 2003. The evolution of the cortico-cerebellar complex in primates: anatomical connections predict patterns of correlated evolution. J Hum Evol. 44:3–10.

Yarkoni T, Poldrack RA, Nichols TE, Van Essen DC, Wager TD. 2011. Large-scale automated synthesis of human functional neuroimaging data. Nat Methods. 8:665–670.
